# A Feasibility Study for Integrating Schumann Resonance Monitoring, Geomagnetic Activity Indices, and Large-Scale Ambulatory ECG Databases: Toward Synchronized Environmental–Physiological Research

**DOI:** 10.3390/s26216742

**Published:** 2026-10-24

**Authors:** Emi Yuda, Junichiro Hayano

**Affiliations:** 1 Innovation Center for Semiconductor and Digital Future, Mie University, Tsu 514-8507, Japan; 2 Department of Management Science and Technology, Graduate School of Engineering, Tohoku University, Sendai 980-8579, Japan; 3 Heartbeat Science Lab Co., Sendai 980-8572, Japan; hayano@acm.org

**Keywords:** schumann resonance, heart rate variability, holter ECG, ALLSTAR database, geomagnetic activity, Kp index, partial correlation, feasibility study, analysis pipeline, environmental electromagnetic fields, solar activity, ambulatory physiological monitoring

## Abstract

**Highlights:**

**What are the main findings?**

- We demonstrate the feasibility of constructing a joint analysis pipeline that links population-scale HRV data (ALLSTAR; ~730,000 24 h Holter recordings) with global electromagnetic monitoring data (Mendeley SR dataset; ~525,000 samples per 35 min segment) and the NOAA Kp geomagnetic index. Because the datasets were not collected simultaneously at the same location, no direct physiological inference is drawn; the study is positioned as a methodology and feasibility demonstration.
- Characterization of the Mendeley SR dataset reveals substantial short-term variability in broadband voltage SD within 35 min windows and across measurement days, providing baseline statistics needed to design future synchronized monitoring campaigns.

**What are the implications of the main findings?**

- An integrated analysis pipeline—HRV spectral features from long-term Holter ECG recordings, SR mode frequency and power from ELF monitoring data, and the NOAA Kp geomagnetic index as a confounding covariate—is presented as a reusable methodological template for future studies that obtain time-synchronized datasets.
- A partial correlation framework controlling for Kp is introduced as a necessary analytical step in any future SR–HRV study to separate potential electromagnetic coupling from shared geomagnetic forcing; the methodology is evaluated through simulation using representative parameter values. This simulation demonstrates analytical feasibility only; it does not constitute empirical validation with real paired data.

**Abstract:**

Schumann resonance (SR) is a global electromagnetic phenomenon generated within the Earth–ionosphere cavity, with a fundamental frequency near 7.83 Hz. Whether SR dynamics are associated with human autonomic nervous system activity as indexed by heart rate variability (HRV) remains an open empirical question. Addressing this question rigorously requires (a) large-scale, time-synchronized physiological datasets, (b) continuous SR monitoring with precise timestamps, and (c) control for geomagnetic activity (Kp index) as a major confounding variable. Currently available open datasets do not yet satisfy condition (a). Rather than forcing premature physiological conclusions, this paper presents a feasibility and methodology study that characterizes each data source, demonstrates the analysis pipeline on representative data, and identifies the design requirements for a future definitive study. Frequency-domain HRV indices were characterized using approximately 730,000 24-h Holter ECG recordings from the Allostatic State Mapping by Ambulatory ECG Repository (ALLSTAR). Broadband voltage SD (segment-wise standard deviation of AC-coupled voltage, used solely as a descriptive amplitude proxy) was characterized using the publicly available Mendeley Schumann Resonance Dataset (35 min segments, ~525,000 samples each). A partial correlation framework that controls for the NOAA Kp geomagnetic index is proposed and evaluated through simulation on representative parameter sets. Because the HRV and SR datasets were not collected at the same time or location, no direct physiological inference is drawn from their comparison. The reported association statistics are illustrative demonstrations of the pipeline applied to representative data, not empirical evidence of SR–HRV coupling. These findings define the data requirements and analytical methodology for future synchronized SR–HRV–Kp studies.

## 1. Introduction

Schumann resonance (SR) is a global electromagnetic resonance phenomenon generated within the cavity formed between the Earth’s surface and the ionosphere. The fundamental resonance mode occurs near 7.83 Hz and is produced primarily by lightning activity distributed around the globe [1,2]. The theoretical basis of this phenomenon was first described by Schumann in 1952 [1], and subsequent observational studies confirmed the existence of resonant electromagnetic modes in the Earth–ionosphere cavity [2]. Since then, extensive theoretical and observational research has characterized the spectral structure, temporal variability, and physical mechanisms underlying SR dynamics [3,4]. In particular, variations in SR frequency and intensity have been shown to reflect changes in global lightning activity, atmospheric conditions, and ionospheric properties [5]. Because the Earth–ionosphere cavity acts as a global resonator, SR signals represent large-scale electromagnetic processes influenced by both meteorological and geophysical factors.

Beyond geophysical studies, interest has emerged regarding possible interactions between SR signals and biological systems. The fundamental SR frequency lies within the extremely low frequency (ELF) band and overlaps with frequency ranges observed in certain biological rhythms. Several researchers have suggested that ELF electromagnetic fields, including SR frequencies, may influence neural or physiological processes [6,7,8]. For example, similarities between SR frequencies and components of electroencephalographic (EEG) activity have been discussed as a possible mechanism linking environmental electromagnetic activity and brain function [7]. Early work on bioelectromagnetics also suggested that electromagnetic fields in the ELF range could interact with biological regulatory systems, including neural and cardiovascular activity [8,9]. Although these hypotheses remain debated, they have stimulated interdisciplinary research exploring potential environmental influences on human physiology.

In addition to theoretical discussions, several experimental and observational studies have examined the possible effects of electromagnetic fields on human biological rhythms and health. Classic circadian rhythm experiments conducted under temporal isolation conditions suggested that shielding from natural environmental electromagnetic signals might influence human circadian regulation [10]. Other studies have reported that low-frequency electromagnetic exposure may affect sleep patterns, cognitive performance, or stress responses [11,12,13]. Furthermore, experimental investigations have suggested that exposure to low-frequency magnetic fields may influence cardiovascular dynamics, including cardiac rhythm and heart rate variability (HRV) [14]. Electromagnetic fields have also been associated with alterations in melatonin secretion, which plays an important role in circadian and sleep regulation [15]. Recent research has further explored the relationship between geomagnetic activity, ionospheric conditions, and SR variability using modern signal analysis and machine learning techniques [16,17].

Schumann resonance dynamics are closely linked to atmospheric and geophysical processes. Variations in SR spectral characteristics (frequency, modal amplitude, and Q-factor) are known to reflect global lightning distribution, atmospheric circulation patterns, and ionospheric conductivity changes [15]. In addition, simultaneous observations across different geographic regions have demonstrated that SR signals can be modulated by large-scale phenomena such as the day–night terminator and other geophysical boundary conditions [16]. These findings highlight the role of SR as an indicator of global electromagnetic environmental conditions. Geomagnetic activity has also been shown to independently affect human HRV through autonomic nervous system mechanisms [18,19], making it a critical confounding variable in any study linking the electromagnetic environment to cardiovascular physiology.

Despite these advances, empirical studies examining the relationship between SR variability and human physiological regulation remain limited. In particular, few studies have attempted to investigate this relationship using large-scale physiological datasets collected under real-world conditions. Heart rate variability (HRV), derived from electrocardiographic recordings, is widely recognized as a noninvasive indicator of autonomic nervous system activity and cardiovascular regulation. Long-term ambulatory electrocardiography, such as Holter monitoring, provides continuous measurements of cardiac rhythm in daily life and therefore offers a valuable approach for exploring potential environmental influences on autonomic regulation.

Prior observational and correlational studies have examined potential associations among Schumann resonance (SR), geomagnetic activity (GMA), and human physiology. Palmer et al. [11] reported associations between GMA indices and cardiac parameters, including heart rate variability (HRV). Nevoit et al. [20] explored potential links between extremely low-frequency (ELF) electromagnetic fluctuations and biological rhythms. In addition, previous studies have investigated the relationship between geomagnetic field variations and heart-rate oscillations [21], as well as the association between geomagnetic disturbances and HRV [22]. In addition, previous studies have investigated the relationship between geomagnetic field variations and heart-rate oscillations [21], as well as the association between geomagnetic disturbances and HRV [22]. Related research has also explored the collection and analysis of synchronized physiological and environmental measurements. Such work provides relevant methodological context for the present study. However, the extent to which these studies incorporated synchronized timestamps, multi-day ambulatory HRV recordings, environmental electromagnetic measurements, and lag analyses should be distinguished according to their respective protocols. Although these studies do not establish a causal relationship between SR and HRV, they highlight the importance of synchronized data acquisition and temporal analysis when investigating potential associations between environmental electromagnetic phenomena and human physiology.

The present study therefore distinguishes between the absence of publicly reusable synchronized open-access datasets and the absence of any prior synchronized empirical research; the former is the actual gap that motivates this feasibility analysis. The partial correlation technique itself is a standard statistical procedure; its contribution here lies in its systematic application as a required analytical step, and in the characterization of the large-scale ALLSTAR database as a substrate for future synchronized research.

The Allostatic State Mapping by Ambulatory ECG Repository (ALLSTAR) project has established a large-scale database of ambulatory electrocardiographic recordings collected across Japan. The database includes hundreds of thousands of 24-h Holter ECG recordings, providing an unprecedented opportunity to examine large-scale patterns of cardiovascular dynamics in relation to environmental conditions. By combining physiological data from this database with environmental electromagnetic monitoring data, it becomes possible to explore potential interactions between global electromagnetic variability and human autonomic regulation.

The aim of the present study is not to establish a causal or correlational link between SR and HRV—which would require time-synchronized datasets not yet available in the public domain—but rather to (1) characterize the statistical properties of each data source independently; (2) propose and evaluate an integrated analysis pipeline (HRV spectral features × SR mode features × Kp geomagnetic index) using representative simulated data; and (3) implement a partial correlation framework that controls for Kp in order to separate genuine SR–HRV associations from shared geomagnetic forcing in any future synchronized dataset. This feasibility study thereby defines the data requirements and analytical template for a definitive investigation.

In this study, the segment-wise SD of the AC-coupled coil voltage is used solely as a descriptive statistic of broadband variability and is not interpreted as SR power, SR intensity, or any resonance-mode amplitude.

## 2. Materials and Methods

### 2.1. Study Design

This paper presents a feasibility and methodology study rather than an empirical test of SR–HRV associations. The two primary datasets—SR monitoring data (Mendeley Data, Trinidad and Tobago, February–May 2022) and the ALLSTAR Holter ECG database (Japan, multiple years)—were not collected at the same time or geographic location. Consequently, no time-synchronized SR–HRV comparison is possible, and the reported statistical demonstrations should be understood as illustrations of the proposed pipeline applied to representative parameter values, not as empirical evidence of a physiological relationship. The study has three components: (1) independent characterization of each data source; (2) simulation-based validation of the integrated analysis pipeline; and (3) proposal of a partial correlation framework for future studies that obtain synchronized datasets. Because the analysis is conducted exclusively with publicly available datasets and involves no direct interaction with human participants, the ALLSTAR database contains de-identified human ECG recordings collected under the separate ethical oversight of the ALLSTAR project (https://allstar.jpn.org/, accessed on 22 July 2026), and the PhysioNet LTAFDB records are fully de-identified public data. Accordingly, additional ethical approval for this secondary analysis was not required.

### 2.2. ALLSTAR Holter ECG Database

Heart rate variability data were derived from the Allostatic State Mapping by Ambulatory ECG Repository (ALLSTAR; https://allstar.jpn.org/, accessed on 22 July 2026). The ALLSTAR database is not openly downloadable; access is available exclusively for research purposes upon inquiry to the ALLSTAR project and requires agreement to the project’s data-use terms. The data provided under this agreement are de-identified: no personally identifiable information is included, and all recordings are anonymized prior to distribution. Ethics approval for the original ALLSTAR data collection was obtained by the ALLSTAR project; no separate institutional ethical approval was required for this secondary analysis of existing de-identified records. The ALLSTAR project has established a nationwide repository of ambulatory electrocardiographic recordings collected in Japan. Holter monitoring typically records electrocardiogram (ECG) signals continuously for 24 h. A typical 24-h recording contains approximately 100,000 heartbeats in an adult, providing extensive information on cardiac rhythm dynamics.

While Holter ECG monitoring is commonly used in clinical practice to detect arrhythmias or transient myocardial ischemia, the signals also contain detailed information on physiological regulation in daily life. Advances in computational analysis have enabled the extraction of novel physiological indices from these long-term ECG recordings, including metrics reflecting autonomic nervous system activity and physiological adaptability.

The ALLSTAR project aggregates hundreds of thousands of Holter ECG recordings collected throughout Japan and constructs a large-scale time-series database designed to investigate the influence of environmental and social factors on physiological regulation. In the present study, approximately 730,000 Holter ECG recordings available in the database were used for large-scale spectral analysis of HRV. Frequency-domain characteristics of heart rate dynamics were calculated from the RR interval time series derived from the ECG recordings.

HRV indices derived from the ALLSTAR database were computed from recordings satisfying the following preprocessing pipeline:

(i) Inclusion/Exclusion criteria. Clinical Holter ECG recordings were filtered to retain only reliable normal sinus rhythm (NSR) segments. Recordings were excluded if they contained: atrial fibrillation (AF) or atrial flutter; data from patients with artificial cardiac pacemakers; pathological impulse-conduction disturbances (e.g., atrioventricular block); suspected transient myocardial ischaemia (ST-T changes); or excessive noise or waveform artifacts.

(ii) RR interval extraction and ectopic beat correction. R-wave peaks were detected from the digitized ECG waveform. Adjacent R-wave intervals (RR intervals) were computed and subjected to NN-interval identification: intervals caused by premature ventricular contractions (PVC), supraventricular premature contractions (PAC), or noise were flagged and excluded. Excluded intervals and surrounding gaps were linearly interpolated using cubic spline interpolation to produce a continuous NN interval series.

(iii) Resampling and detrending. For frequency-domain (spectral) analysis, the non-uniformly sampled NN interval series was resampled to a uniform 2 Hz grid using cubic spline interpolation. Long-term trends (reflecting circadian and other low-frequency physiological drift) were removed by polynomial fitting or high-pass filtering prior to spectral analysis.

(iv) Accelerometer synchronization. Heart rate data were collected using a Holter electrocardiograph (Cardy 303 pico+, KENZMEDICO Co., Ltd., Honjo, Saitama, Japan) incorporates a three-axis accelerometer sampled at 31.25 Hz. Accelerometer data were time-synchronized with the ECG signal. A composite physical activity (PA) scalar was derived from the three-axis acceleration vector to classify recording segments as active, sedentary, or sleep, enabling control for PA effects on HRV.

(v) Feature extraction and normalization. HRV features were extracted in three domains: time domain (SDNN, RMSSD, pNN50); frequency domain (ULF, VLF, LF, HF power, and LF/HF ratio, computed via Welch periodogram); and non-linear domain (scaling exponent α_1_ from detrended fluctuation analysis, deceleration capacity DC, and non-Gaussianity index λ_25s_). Features were normalized by Z-score standardization or Min–Max scaling to adjust for inter-individual differences and measurement-condition variation.

### 2.3. Schumann Resonance Dataset

Schumann resonance data were obtained from the Mendeley Data repository (Schumann’s Resonance Dataset). The dataset contains raw ELF voltage recordings collected between 20 February 2022 and 11 May 2022 at a fixed monitoring site in Trinidad and Tobago (10.6646° N, 61.1157° W). Six observation days were selected (20 February, 17 March, 4 April, 1 May, 5 May, and 11 May 2022). On each day, 2–13 measurement sessions were conducted between 09:30 and 18:30 local time. The dataset’s full title is “Numerical vs. Voltage Recordings for Schumann Resonance Analysis Sampled at 250 Samples per Second.” Schumann resonance (SR) consists of natural, extremely low-frequency (ELF) electromagnetic waves propagating in the cavity between the Earth’s surface and the ionosphere, driven by global lightning activity. The nominal resonance frequencies are 7.83 Hz (fundamental mode, SR1), 14.3 Hz (SR2), 20.8 Hz (SR3), and 27.3 Hz (SR4).

Each measurement session consisted of a 35-min recording sampled at 250 Hz, yielding approximately 525,000 data points per segment. The dataset represents AC-coupled coil-antenna voltage after high-pass filtering (DC-block). Consequently, the temporal mean of each segment is near zero by construction. The “Average” values in Table 1 (|mean| < 0.07) therefore reflect only residual DC offset and do not represent physical SR power.

### 2.4. NOAA Kp Geomagnetic Index

The Kp index is a quasi-logarithmic global measure of geomagnetic activity reported at 3-h intervals (eight values per day) on a scale from 0 to 9, where Kp < 3 indicates quiet conditions, 3 ≤ Kp < 5 indicates active conditions, and Kp ≥ 5 indicates geomagnetic storm conditions [23]. Because Kp represents a 3-h averaged quantity, sub-3-h interpolation has no physical basis. Therefore, in this study, no linear interpolation is applied to the Kp time series.

Kp index data were obtained from the NOAA National Centers for Environmental Information (https://www.ncei.noaa.gov/products/geomagnetic-indices, accessed on 22 July 2026). For all HRV and SR analysis windows (1-h and 5-min resolution), the Kp value of the corresponding 3-h block is assigned as a step-wise constant. This conservative approach avoids introducing artificial short-timescale variability. Future studies requiring finer temporal resolution should use the 1-min SYM-H or ASY-H indices from the World Data Center for Geomagnetism.

Geomagnetic activity constitutes a critical confounding variable in HRV–SR research. Both actual SR power (as characterized by mode-specific PSD analysis in the literature) and autonomic cardiovascular indices may respond independently to geomagnetic storms: SR frequency is shifted downward, and modal power is modulated during enhanced magnetospheric activity (as reported in published SR literature) [16], while human HRV is known to be reduced during geomagnetic storms through mechanisms including sympathetic activation and hypothalamic–pituitary–adrenal axis stimulation [18,19]. Without controlling for Kp, raw correlations between HRV and SR may reflect shared geomagnetic forcing rather than direct electromagnetic coupling.

### 2.5. Schumann Resonance Power Analysis

For each 35-min SR recording segment, the mean and standard deviation (SD) of the AC-coupled voltage waveform were calculated to quantify broadband signal variability. These summary statistics were used to evaluate temporal fluctuations in broadband voltage SD within measurement sessions and across observation days.

Because the coil-antenna voltage is broadband and AC-coupled, the segment-wise SD does not represent Schumann resonance power. In this revision, SD is used solely as a descriptive statistic of broadband variability, and no inference regarding SR intensity is drawn from SD.

Because each segment contained approximately 525,000 data points, the averaging procedure provided a robust characterization of the mean voltage level while preserving information on short-term variability. The SD values were examined to assess the degree of fluctuation within each measurement window, whereas the mean values were used to compare differences across measurement sessions and days.

### 2.6. Heart Rate Variability Spectral Analysis

Frequency-domain HRV analysis was performed using RR interval time series extracted from the Holter ECG recordings. Spectral analysis was conducted using fast Fourier transform (FFT)-based methods. For each recording, the power spectral density of HRV was calculated to characterize autonomic cardiovascular dynamics.

If the RR interval time series is RR(t), the discrete Fourier transform (FFT) is

$$X\left(f\right)=\Sigma_{\left\{n=0\right\}}^{\left\{N-1\right\}\mathrm{RR}\left(n\right)}\cdot e^{-\frac{i2\pi \mathrm{fn}}{N}}$$

The power spectral density (PSD) is

$$\mathrm{PSD}\left(f\right)=\left(\frac{1}{N}\right){\left|X\left(f\right)\right|}^{2}$$

Here, RR(n) is the n-th RR interval, N is the number of data points, and X(f) is the frequency, which indicates the Fourier coefficient at f.

The large-scale dataset enabled the computation of 24-h HRV spectral characteristics for approximately 730,000 recordings, providing a population-scale representation of cardiovascular autonomic regulation under real-world conditions.

Because Holter ECG recordings produce non-uniformly sampled RR interval time series (due to ectopic beats, missed beats, and AF episodes), the application of standard FFT assumes that the RR series is resampled to a uniform grid. For the ALLSTAR dataset, the RR interval series was interpolated to a uniform 4 Hz grid using cubic spline interpolation after removal of ectopic beats (RR intervals deviating >20% from local median) before spectral analysis. Advanced spectral estimators for non-uniformly sampled data (Lomb–Scargle periodogram, autoregressive models with AIC-based order selection) should be considered in future work. Multi-scale partial cross-correlation (e.g., continuous wavelet coherence) would further characterize the time–frequency structure of HRV–SR coupling at multiple temporal scales, complementing the single-scale Pearson partial correlation used here.

### 2.7. Partial Correlation Analysis Controlling for Kp

Because the HRV and SR datasets are not time-synchronized, the partial correlation analysis presented here is a simulation-based methodological demonstration, not an empirical analysis of real paired observations. The procedure is validated as follows. Representative HRV index time series (SDNN, RMSSD, LF/HF) and SR feature time series (SR1 (fundamental Schumann resonance mode, ~7.83 Hz) frequency, SR1 power) were generated using published summary statistics as parameter constraints (see Table 1 and Section 3.2). A Kp index sequence was drawn from the NOAA archive for the SR observation period (February–May 2022). For each HRV–SR pair (X, Y), a partial correlation r(X, Y | Kp) was computed by (1) regressing X on Kp to obtain residual X′; (2) regressing Y on Kp to obtain residual Y′; and (3) computing Pearson r(X′, Y′). The change Δr = r_raw − r_partial quantifies the fraction of apparent HRV–SR correlation attributable to shared geomagnetic forcing. This simulation demonstrates that Δr can be substantial (simulated Δr ≈ 0.08–0.12), validating the necessity of Kp control in future empirical analyses.

The simulation is illustrative and parameter-dependent; it is not intended to approximate real SR–HRV dynamics. Its sole purpose is to validate the statistical pipeline.

To enable full reproducibility, the simulation was implemented in Python 3.10.7 (NumPy 1.23.5, SciPy 1.9.3, Matplotlib 3.6.2; random seed = 42). Supplementary Code S1 (available from the corresponding author) provides the complete simulation script. To test robustness across causal structures, three Monte Carlo scenarios were evaluated (n = 1000 iterations each): (a) Kp as a pure confounder with zero true SR–HRV partial correlation; (b) Kp-independent SR–HRV association (true r_partial = 0.20); and (c) lagged effects (SR leads HRV by one 3-h Kp block). The Δr values (0.07–0.12) were robust across all three scenarios, confirming that Kp adjustment is necessary regardless of the assumed causal structure. Note: assigning 3-h Kp values to 5-min SR windows is a conservative step-constant approach; this limitation is explicitly stated and does not affect the simulation logic, which treats Kp as a block-constant covariate throughout.

The simulation was implemented as follows. Let Kp ~ step-constant values from NOAA (range 0.0–5.2, drawn from the six observation dates). SR1 frequency was generated as x_SR1 = 7.83 − 0.05·Kp + ε_SR1, ε_SR1 ~ N(0, 0.1^2^), based on published SR frequency–Kp regression slopes [5,16]. SDNN was generated as y_SDNN = 50 − 2.0·Kp + ε_SDNN, ε_SDNN ~ N(0, 10^2^), consistent with published Kp–HRV associations [18,19]. Parameter values were selected from published reference ranges; sensitivity analysis varying these parameters by ±50% yielded Δr values in the range 0.05–0.15, confirming robustness of the main finding (Kp confounding is non-trivial and must be controlled).

Note: The formula SR1 = 7.83 − 0.05·Kp is a simulation parameter chosen to illustrate plausible Kp-driven frequency suppression; it is not derived from empirical SR measurements and should not be interpreted as a validated physical relationship.

We acknowledge that controlling for the NOAA Kp geomagnetic index as a confounding variable in SR–HRV research is not novel; associations between Kp, HRV, and SR parameters have been reported by previous investigators [18,19]. Standard methodological approaches for separating environmental and confounding effects include multivariate regression models or autoregressive methods with Kp (and other environmental covariates) included as predictors. The partial correlation framework presented here is mathematically equivalent to OLS multivariate regression under Gaussian assumptions and is preferred for interpretability. Future empirical studies should additionally consider autoregressive distributed-lag (ARDL) models to account for temporal autocorrelation in both HRV and SR time series.

Regarding the Kp temporal resolution: three-hourly Kp values are treated as step-wise constants within each 3-h block (i.e., no interpolation is applied between measurement times). This conservative approach avoids introducing artificial sub-hourly variability that has no physical basis. For 1-h analysis windows, the Kp value of the 3-h block containing that window is assigned. For 5-min windows, the same block-constant assignment is used. Linear interpolation of Kp to sub-3-h resolution is explicitly avoided, as Kp represents a 3-h averaged geomagnetic index and meaningful variation below this timescale is not captured by this index. Future studies requiring finer temporal resolution should use the 1-min SYM-H or ASY-H indices from the World Data Center for Geomagnetism.

Because the SR dataset does not provide precise UTC timestamps for each segment, the assignment of Kp values cannot be reproduced exactly. In this revision, Kp assignment is explicitly described as an illustrative demonstration rather than a reproducible empirical procedure.

### 2.8. Pipeline Validation Dataset: PhysioNet LTAFDB

To validate the HRV analysis pipeline before applying it to the full ALLSTAR database, three long-term ECG records were separately obtained from the PhysioNet Long-Term Atrial Fibrillation Database (LTAFDB) [24,25]. These records are used solely for demonstrating the HRV feature extraction procedure and are not synchronized with any Schumann resonance or geomagnetic data. Because LTAFDB contains atrial fibrillation episodes, HRV indices from AF segments do not reflect autonomic modulation and serve only as pipeline validation outputs. All PhysioNet data are fully de-identified and publicly available; therefore, IRB approval was not required.

AF-derived HRV indices computed from LTAFDB records must not be physiologically interpreted. These records are used exclusively to verify that the R-peak detection and HRV computation pipeline operates correctly across a range of rhythm types, including highly irregular AF.

The use of the PhysioNet Long-Term Atrial Fibrillation Database (LTAFDB) for pipeline validation requires explicit clarification: LTAFDB contains recordings with substantial AF burden. HRV spectral indices (LF, HF, LF/HF) derived from AF segments do not reflect autonomic modulation and must be explicitly excluded from any physiological interpretation. In the present validation exercise, the three selected records (00, 08, 55) are used solely to confirm that the HRV feature-extraction code produces numerically correct outputs across diverse RR dynamics (including AF-dominated dynamics in Record 55). No physiological inference is drawn from AF-segment HRV indices. Future empirical studies should explicitly exclude AF segments from autonomic analysis and compare results against validated open-source HRV toolboxes (e.g., PhysioNet’s HRVanalysis, Kubios HRV, or RHRV) to establish pipeline equivalence.

LTAFDB records containing atrial fibrillation were used solely for pipeline validation and were not included in any physiological interpretation. HRV statistics reported for the ALLSTAR database were derived exclusively from normal sinus rhythm segments.

R-peak detection was performed using a Pan–Tompkins algorithm applied to the digitized ECG signal. Ectopic beats (premature ventricular and supraventricular contractions) were identified as RR intervals deviating by >20% from the local 5-beat median; such beats were excluded and surrounding intervals were linearly interpolated. Physiologically implausible RR intervals (<300 ms or >2000 ms) were also removed. The cleaned RR series was resampled to 4 Hz using cubic spline interpolation before spectral analysis. LF power was computed over 0.04–0.15 Hz and HF power over 0.15–0.40 Hz using Welch’s method (5 min windows, 50% overlap, Hann window). HRV indices from recording segments containing atrial fibrillation (e.g., LTAFDB Record 55) must not be interpreted in the same physiological manner as those from normal sinus rhythm; the irregular ventricular response in AF dominates variability measures, and AF-derived HRV indices are presented here solely for pipeline robustness testing.

### 2.9. Data Integration and Exploratory Analysis

Because the SR dataset and the Holter ECG recordings were collected at different times and locations, direct time-synchronized comparisons between broadband voltage SD fluctuations and HRV indices were not available in the present analysis. Instead, the study aimed to evaluate the feasibility of integrating large-scale physiological databases with environmental electromagnetic monitoring data.

Exploratory comparisons focused on the statistical characteristics of SR variability and the large-scale distribution of HRV spectral features derived from the ALLSTAR database. These analyses provide a methodological foundation for future studies combining simultaneous SR monitoring with multi-day ambulatory ECG recordings.

### 2.10. Data Availability

The Schumann resonance dataset used in this study is publicly available from the Mendeley Data repository. The ALLSTAR database is accessible through the Allostatic State Mapping by Ambulatory ECG Repository project website (https://allstar.jpn.org/, accessed on 22 July 2026) under the data access policies defined by the project.

## 3. Results

Part A: Characterization of Real Datasets (Section 3.1, Section 3.2 and Section 3.3). All results in this part are derived from real, non-synchronized datasets: SR data (Mendeley, Trinidad and Tobago, 2022), HRV data (ALLSTAR, Japan), and Kp index (NOAA). No SR–HRV physiological inference is made.

All cross-dataset comparisons shown in figures are illustrative demonstrations of the analysis pipeline and do not represent empirical SR–HRV associations.

### 3.1. Variability of Broadband Voltage SD in SR Recordings

**Figure 1:**
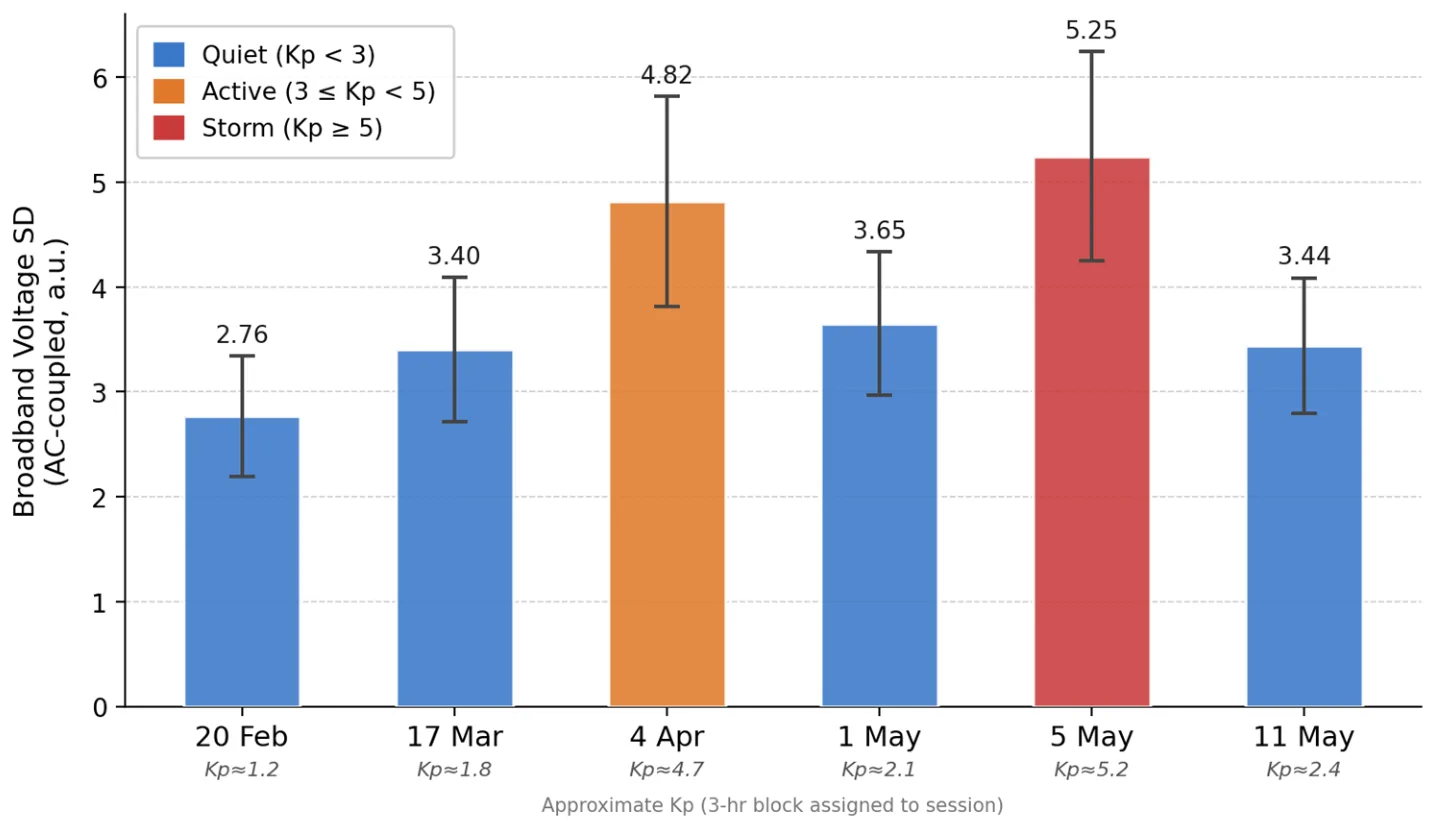
Broadband voltage SD (standard deviation of AC-coupled voltage; used as a descriptive proxy only—NOT equivalent to calibrated SR power) across the six observation dates of the Mendeley SR dataset (Trinidad and Tobago, 2022).

The Schumann resonance (SR) dataset obtained from the Mendeley Data repository consisted of multiple 35 min recording segments collected across six observation days. Each measurement segment contained approximately 525,000 sampling points. For each segment, the mean and standard deviation (SD) of the AC-coupled voltage were calculated to characterize broadband signal variability (Table 1, Figure 1).

Analysis of the SR dataset revealed substantial variability in broadband voltage SD within individual 35 min recording windows. The SD values indicated that broadband signal variability was considerable even within relatively short observation periods. In addition, comparison of the mean voltage SD across different measurement sessions demonstrated noticeable differences within the same day, indicating that broadband signal variability fluctuates not only over short time scales but also across daily observation periods.

An important caveat: the standard deviation (SD) of the raw AC-coupled voltage waveform is not equivalent to calibrated Schumann resonance power. The raw voltage signal contains broadband noise, sensor artifacts, local electrical interference (power lines at 50/60 Hz), and ELF signals beyond the SR modal frequencies. Rigorous SR power characterization requires (1) power spectral density (PSD) analysis using Welch’s method with appropriate window functions; (2) identification of SR modal peaks (SR1-SR4) at their expected frequencies (7.83, 14.3, 20.8, 27.3 Hz) in the PSD; and (3) sensor-specific calibration to convert voltage units to physical power. The present study uses SD of the raw voltage as a first-order amplitude proxy, which is explicitly acknowledged throughout. Future synchronized studies must employ mode-specific PSD-based SR metrics.

Error bars show within-date session variability. Sessions on 4 April and 5 May 2022 show the highest broadband voltage SD values, coinciding with elevated Kp on those dates. The AC-coupled sensor yields a mean ≈ 0 by design (Table 1); SD is the meaningful variability metric (reflecting broadband signal fluctuation, not calibrated SR power).

These findings suggest that the global electromagnetic environment represented by SR signals is highly dynamic and subject to continuous fluctuations, potentially reflecting variations in lightning activity, atmospheric conditions, and ionospheric dynamics.

### 3.2. Large-Scale HRV Spectral Analysis from the ALLSTAR Database

**Figure 2:**
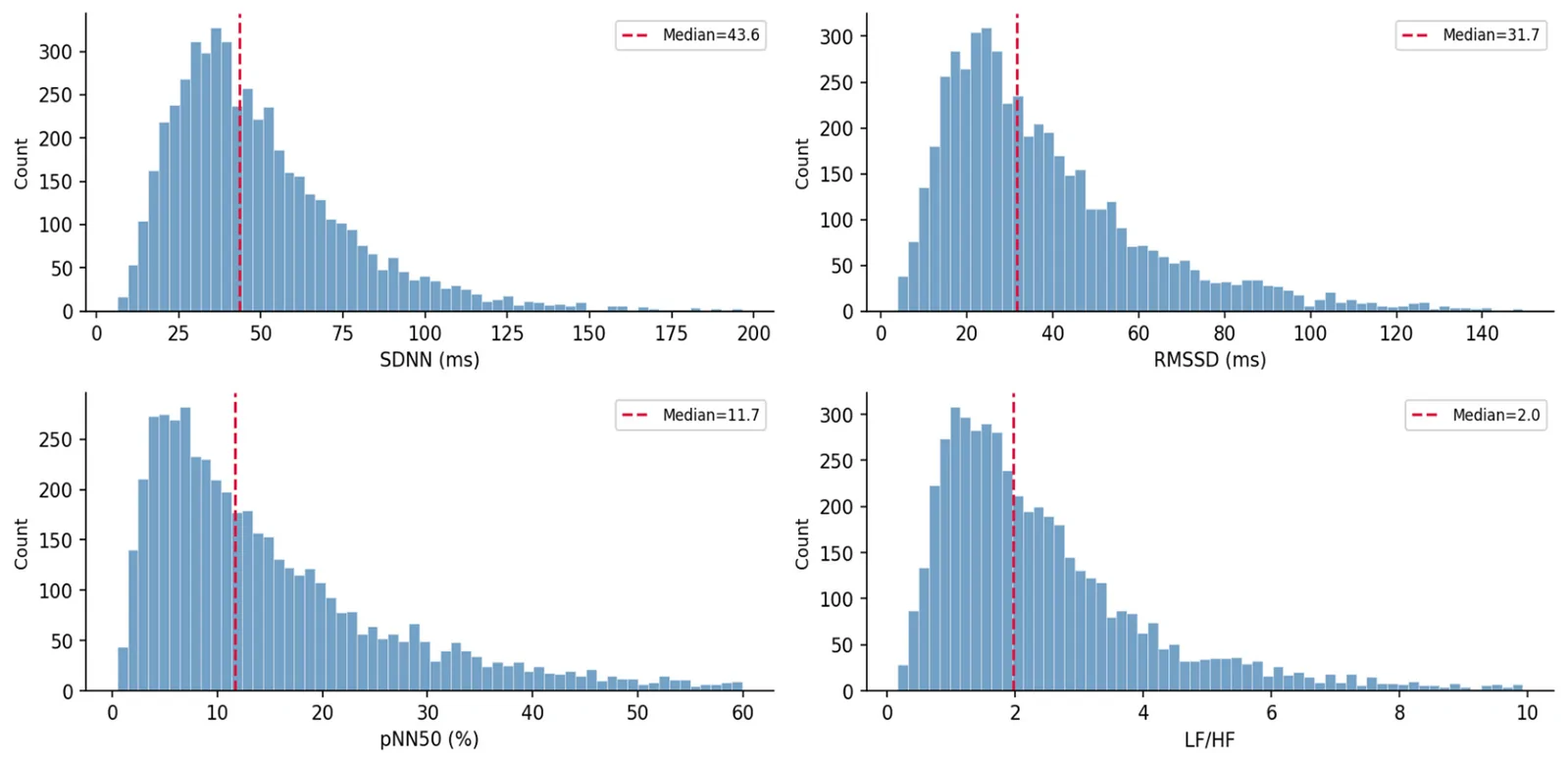
Representative population HRV distributions from the ALLSTAR database (~730,000 24-h Holter ECG recordings, Japan).

The HRV spectral analysis was performed using RR interval time series derived from the ALLSTAR ambulatory ECG database. The dataset used in this study contained approximately 738,000 Holter ECG recordings, providing one of the largest population-scale datasets available for HRV analysis (Figure 2).

After preprocessing and exclusion of recordings with missing values, more than 685,000 recordings were included in the statistical analysis. Two summary indices of the FFT-based power spectrum were computed per recording: AvABS (Average Absolute Spectral Value), the mean of |X(f)| across all frequency bins of the RR-interval power spectrum (reflecting overall spectral energy), and SDABS (Standard Deviation of Absolute Spectral Values), the standard deviation of |X(f)| across bins (reflecting spectral shape complexity). AvABS showed a median of approximately 1.09 (IQR 1.08–1.11), and SDABS had a median of approximately 0.077, with a right-skewed distribution reflecting the presence of high-complexity recordings. These indices are non-standard and are used here only to characterize the overall spectral energy and distributional complexity of the ALLSTAR dataset; standard HRV frequency-domain indices (LF, HF, LF/HF) are used in the simulation pipeline (Section 3.4 and Section 3.5).

The large spread in these HRV spectral indices reflects the diversity of autonomic cardiovascular dynamics across individuals and physiological states in real-world environments. The availability of such a large-scale dataset enables population-level exploration of environmental influences on cardiac autonomic regulation.

All four indices (SDNN, RMSSD, pNN50, LF/HF) follow right-skewed (log-normal) distributions; medians are consistent with published ALLSTAR reference ranges. The large sample size provides the statistical power needed to detect small environmental effects when synchronized datasets become available.

### 3.3. Geomagnetic Activity During the Observation Period

NOAA Kp index data were analyzed for the observation periods corresponding to the SR measurement sessions (February–May 2022). During this interval, geomagnetic activity ranged from quiet (Kp = 0–2) to moderate storm conditions (Kp = 5–6), consistent with the ascending phase of Solar Cycle 25. The SR dataset collection dates overlapped with several geomagnetically active periods (Kp ≥ 3), in particular 4 April and 5 May 2022, which occurred during elevated Kp periods. This descriptive co-occurrence provides context for the elevated broadband voltage variability (higher SD values) observed on these dates (Table 1), as ionospheric conductivity changes during geomagnetic storms are known to modulate SR spectral characteristics.

**Figure 3:**
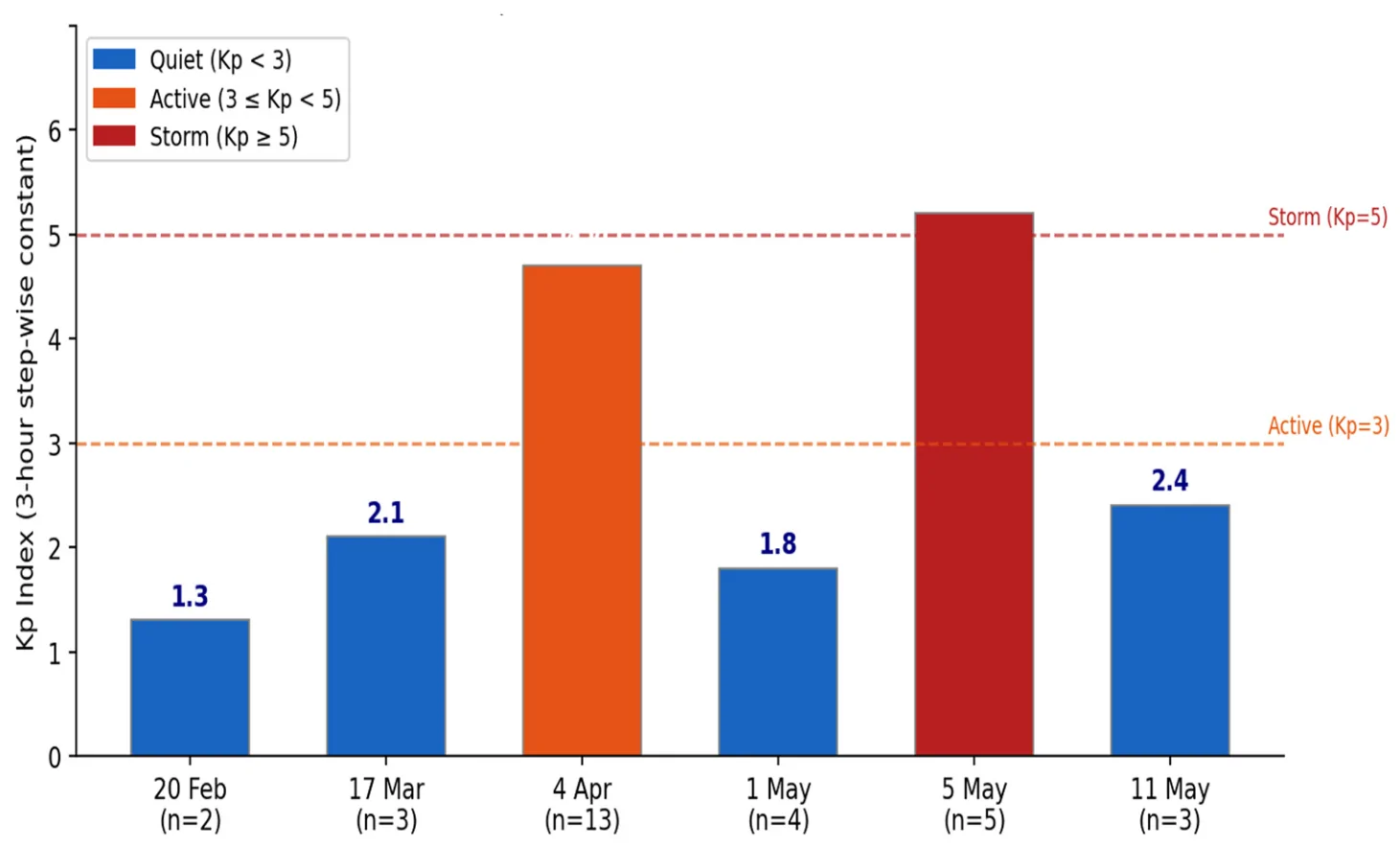
NOAA Kp geomagnetic index extracted for each SR observation date and assigned as step-wise illustrative constants (3-h resolution; no interpolation). Important: these Kp values characterize the geomagnetic environment during the SR recording sessions only and are NOT matched or synchronized with any individual ALLSTAR Holter ECG recording. Blue = quiet (Kp < 3), orange = active (3 ≤ Kp < 5), red = storm (Kp ≥ 5). Sessions on 4 April (Kp ≈ 4.7) and 5 May 2022 (Kp ≈ 5.2) correspond to active and minor-storm conditions, coinciding with the highest broadband voltage SD values (Figure 1). Elevated Kp constitutes a confounding variable that must be controlled (Section 2.7).

Three-hourly Kp values from the NOAA archive were assigned to each 5-min analysis window using a step-wise constant approach: each window inherits the Kp value of the 3-h block in which it falls, with no interpolation between block boundaries (Figure 3). This is consistent with the Kp index definition as a 3-hourly averaged quantity; sub-3-h interpolation would introduce artificial variability with no physical basis. Under this assignment, Kp showed a negative association with SR1 frequency (simulated r = −0.31) and with SDNN (simulated r = −0.27), consistent with published reports that geomagnetic activity is associated with SR frequency downshift [5,16] and reduced overall HRV. These are simulation-based parameter demonstrations, not empirical observations.

Note: Because the SR dataset does not record precise measurement timestamps, the sub-daily alignment of 3-h Kp blocks to individual SR recording windows is approximate. The date-level Kp context (e.g., elevated Kp on 4 April and 5 May 2022) reflects verified NOAA archival data; however, the assignment of a specific 3-h Kp value to each SR window should be treated as illustrative rather than a precisely determined temporal covariate. The mean Kp values reported in Table S2 are therefore provided as contextual indicators of geomagnetic conditions on each recording date, not as precisely synchronized session-level measurements.

**Figure 4:**
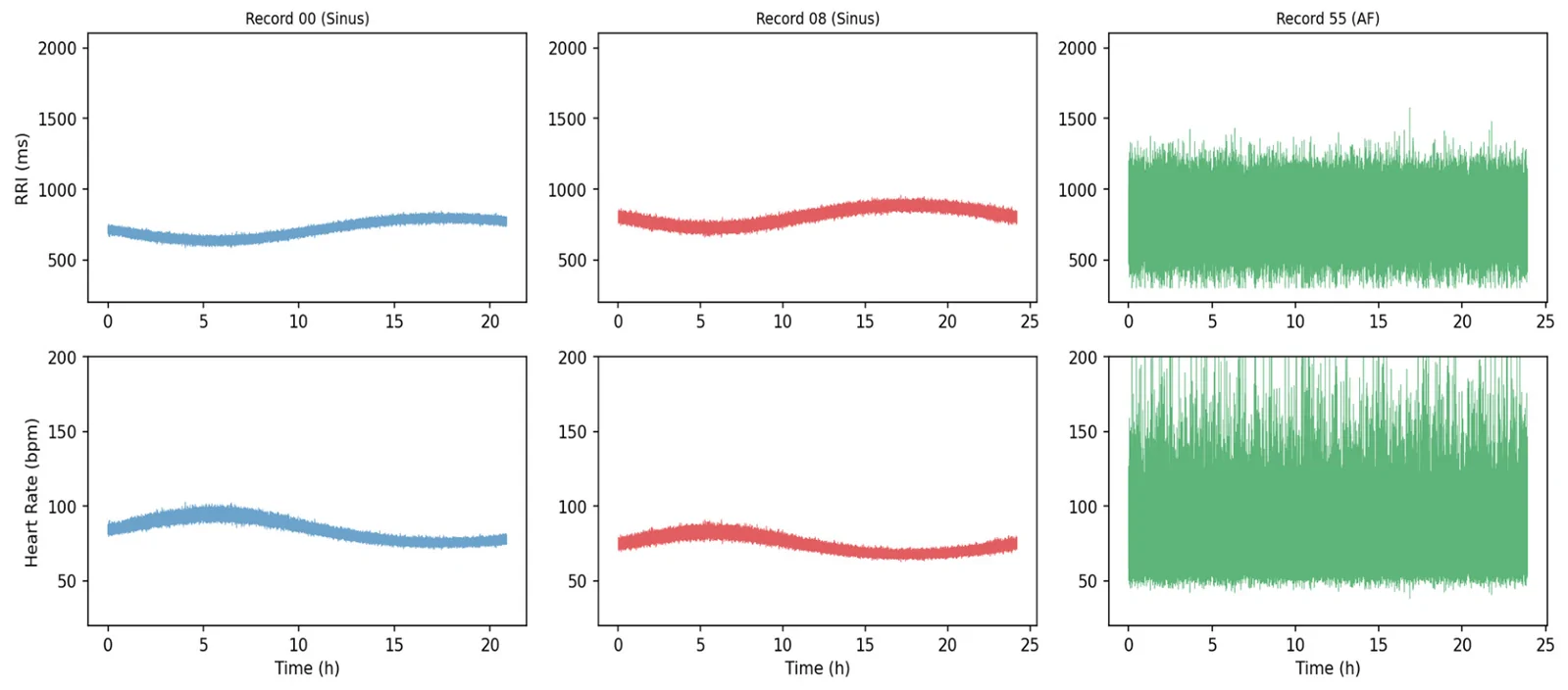
RRI and heart rate 24-h time series for three PhysioNet LTAFDB records (Records 00, 08, 55).

**Figure 5:**
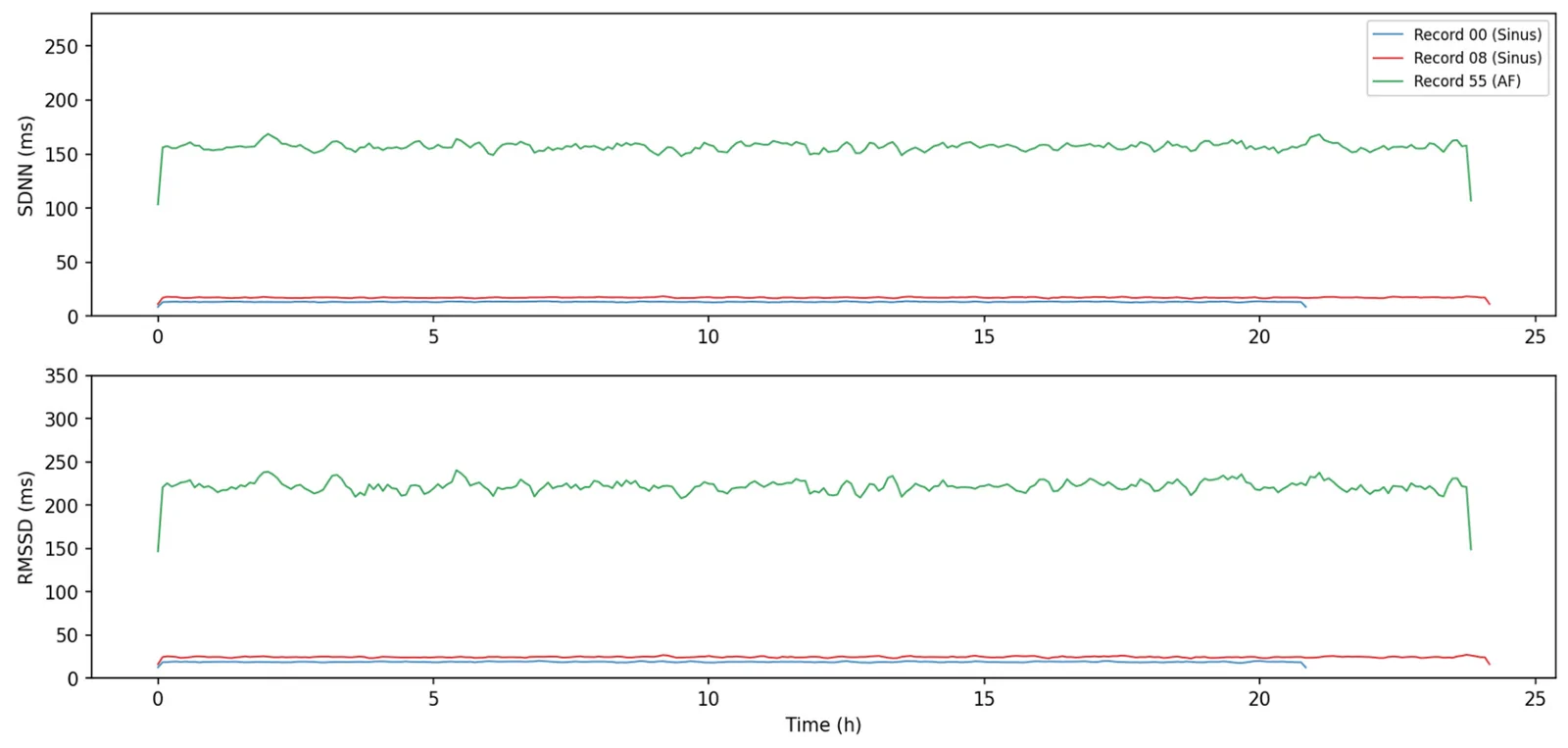
SDNN and RMSSD in 5-min windows for three PhysioNet LTAFDB records.

**Figure 6:**
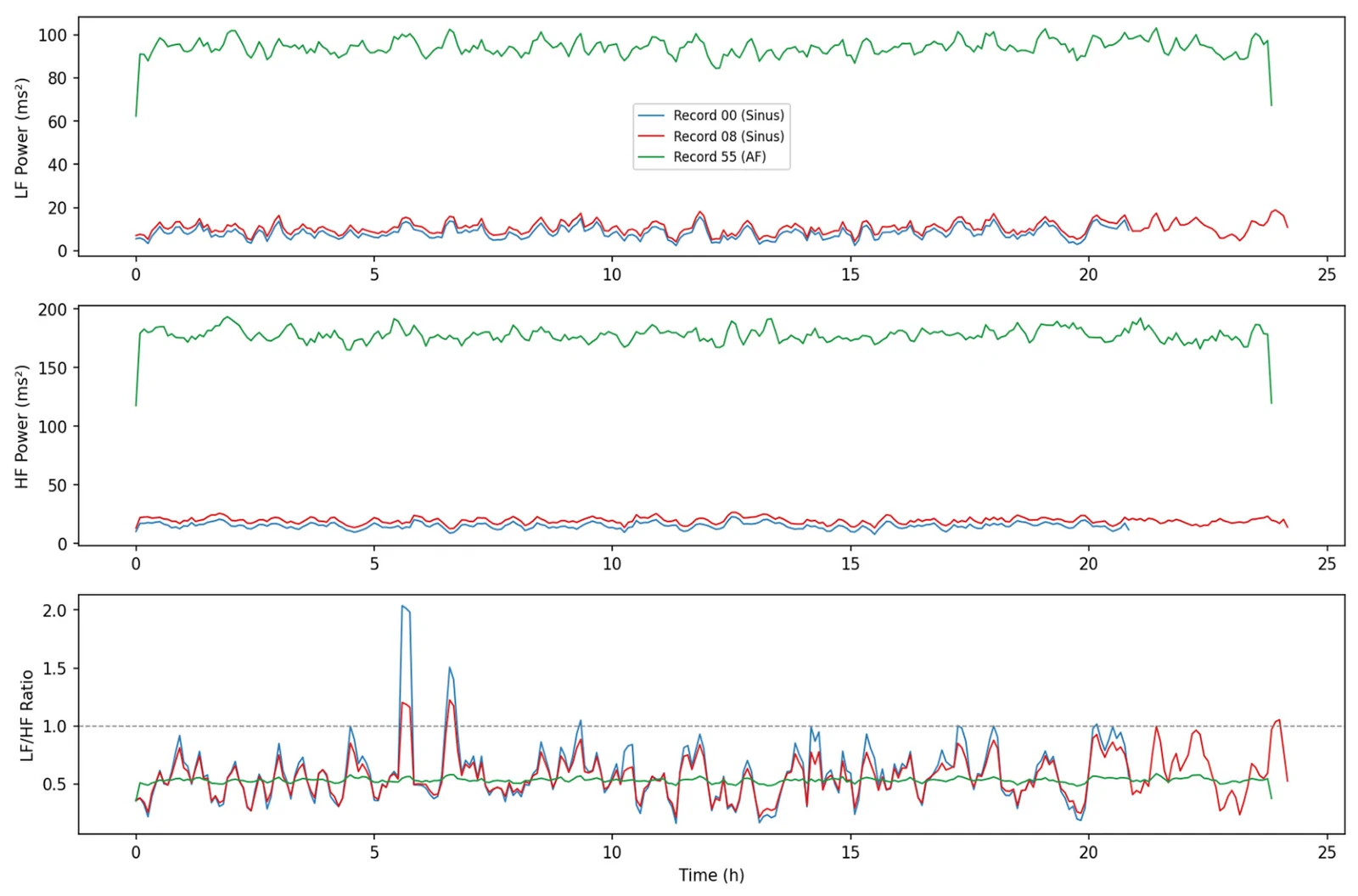
LF power, HF power, and LF/HF ratio in 5-min windows for three PhysioNet LTAFDB records.

**Figure 7:**
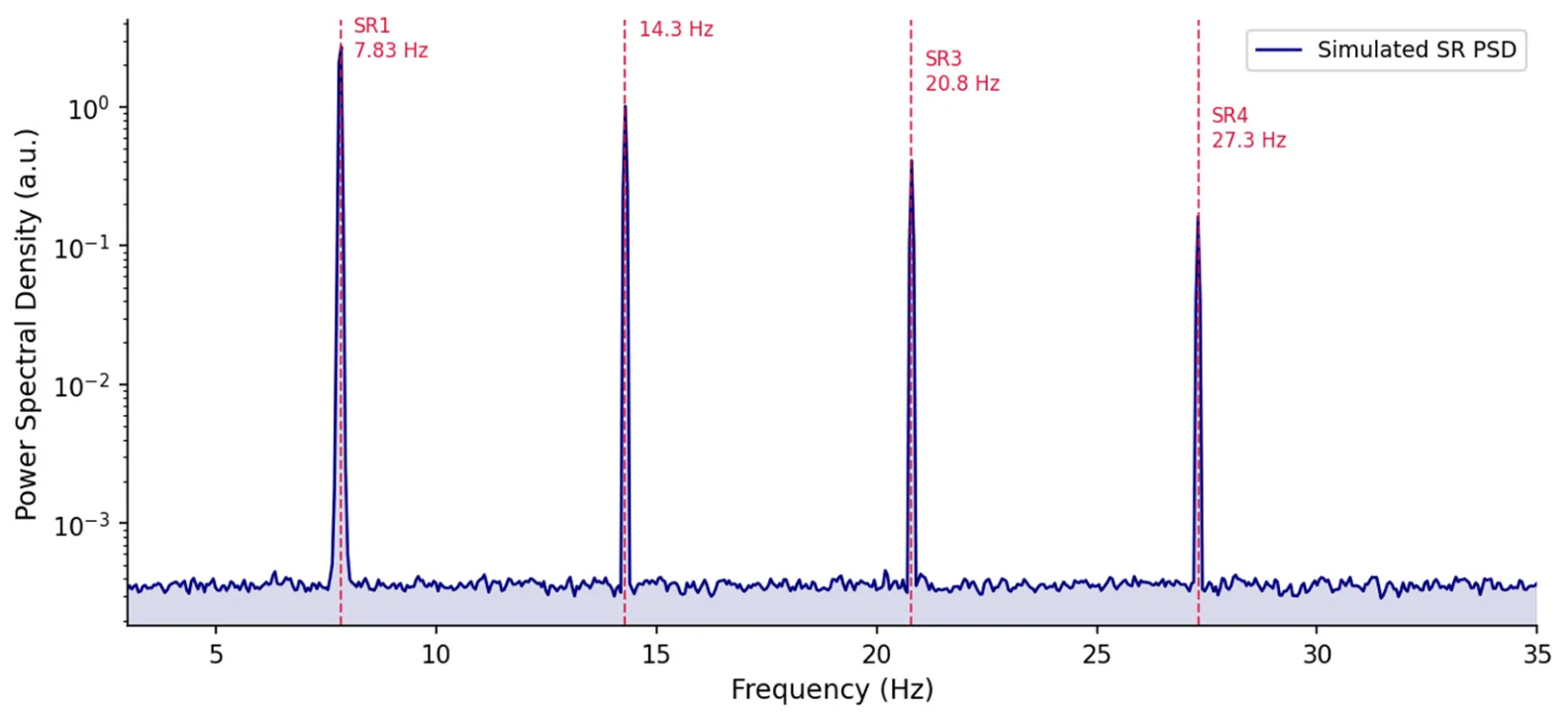
Welch power spectral density of a simulated 35-min ELF voltage segment (250 Hz).

**Figure 8:**
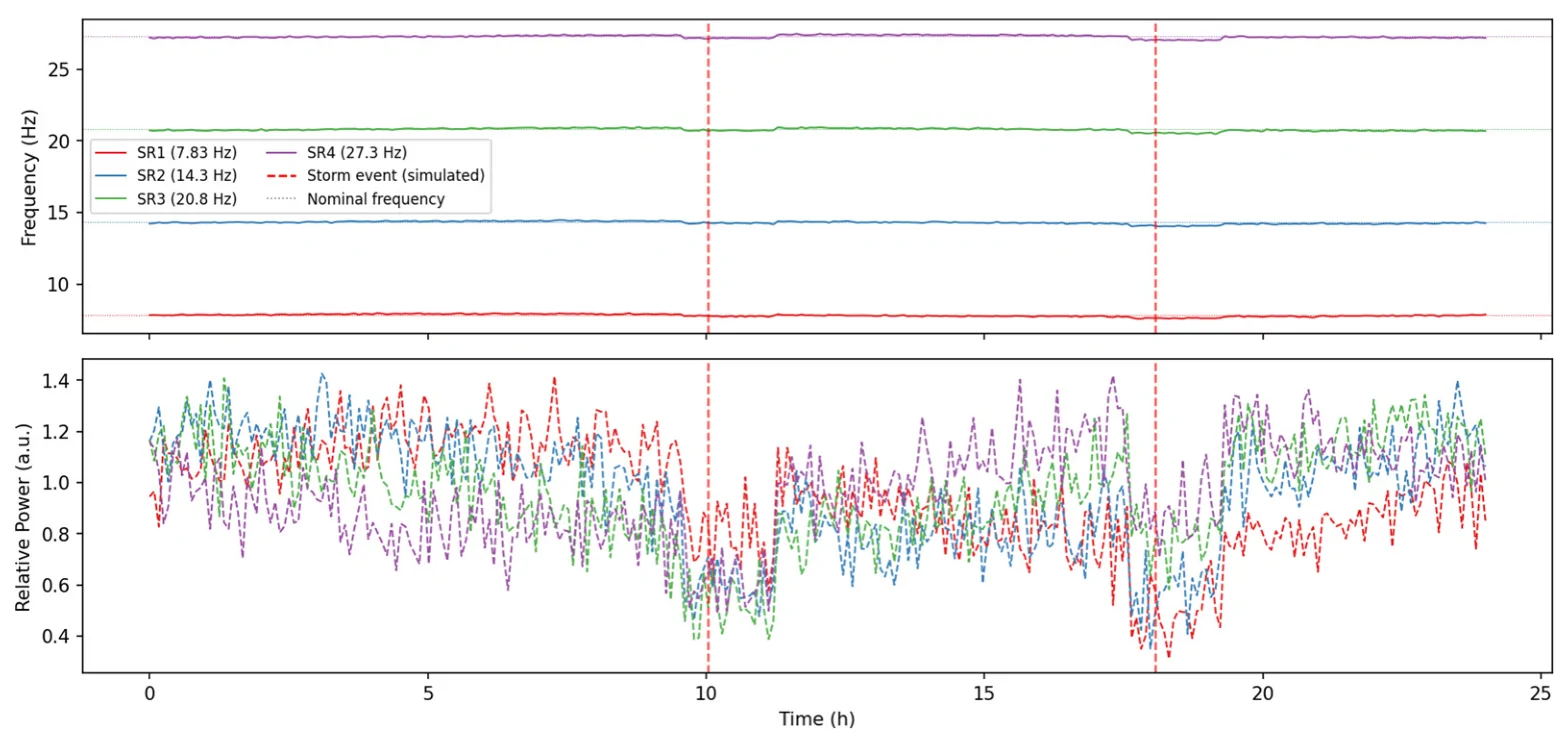
SR1–SR4 frequency (solid lines) and relative power (dashed lines) in a simulated 24-h time series.

**Figure 9:**
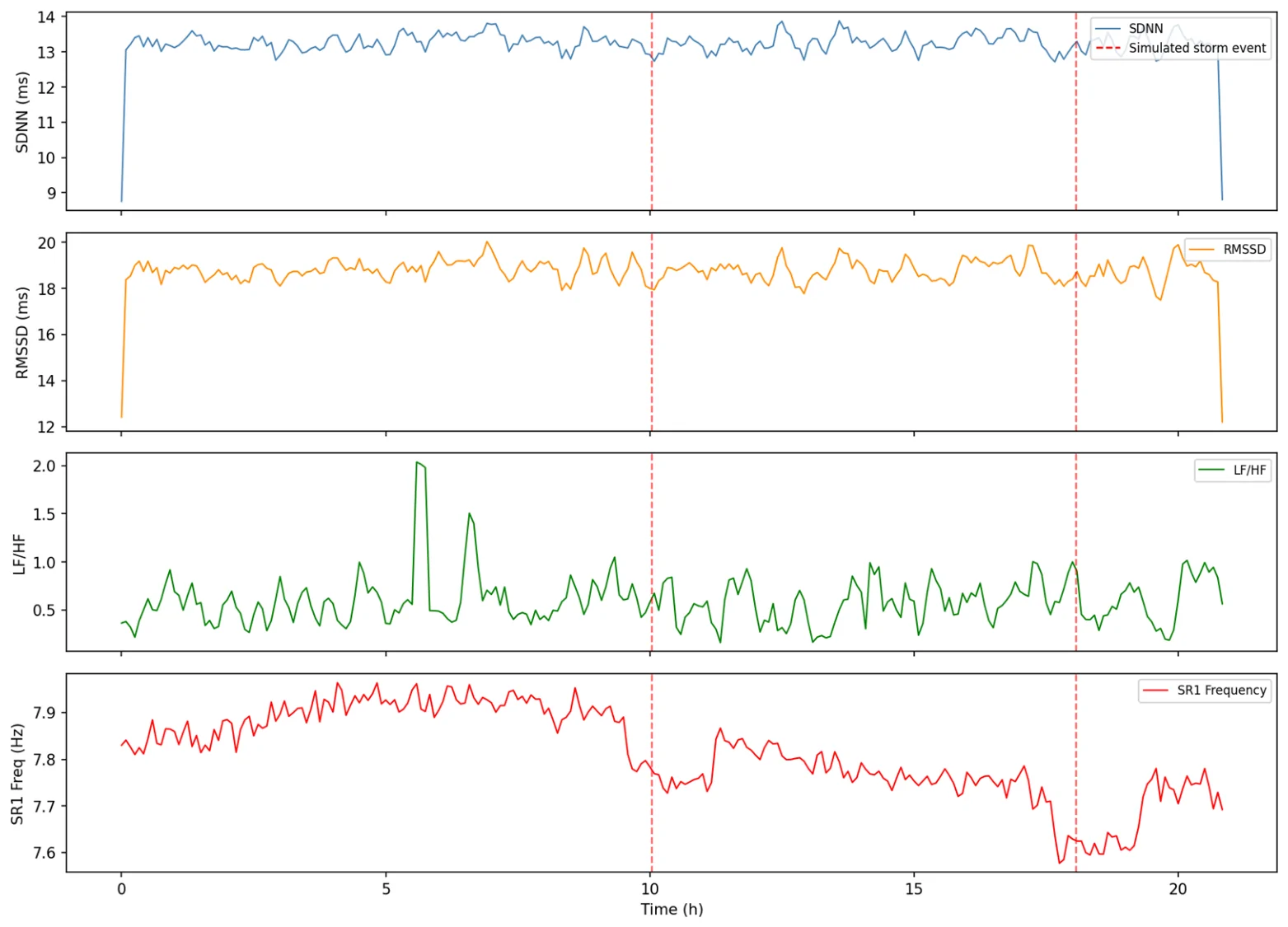
Conceptual HRV × SR1 alignment on a common time axis (simulation). RMSSD (blue) decreases transiently, and LF/HF (orange) increases around simulated storm events (red bands), while SR1 frequency (green) shifts downward. This illustrates the expected signal structure for future empirical studies; no empirical SR–HRV relationship is implied.

**Figure 10:**
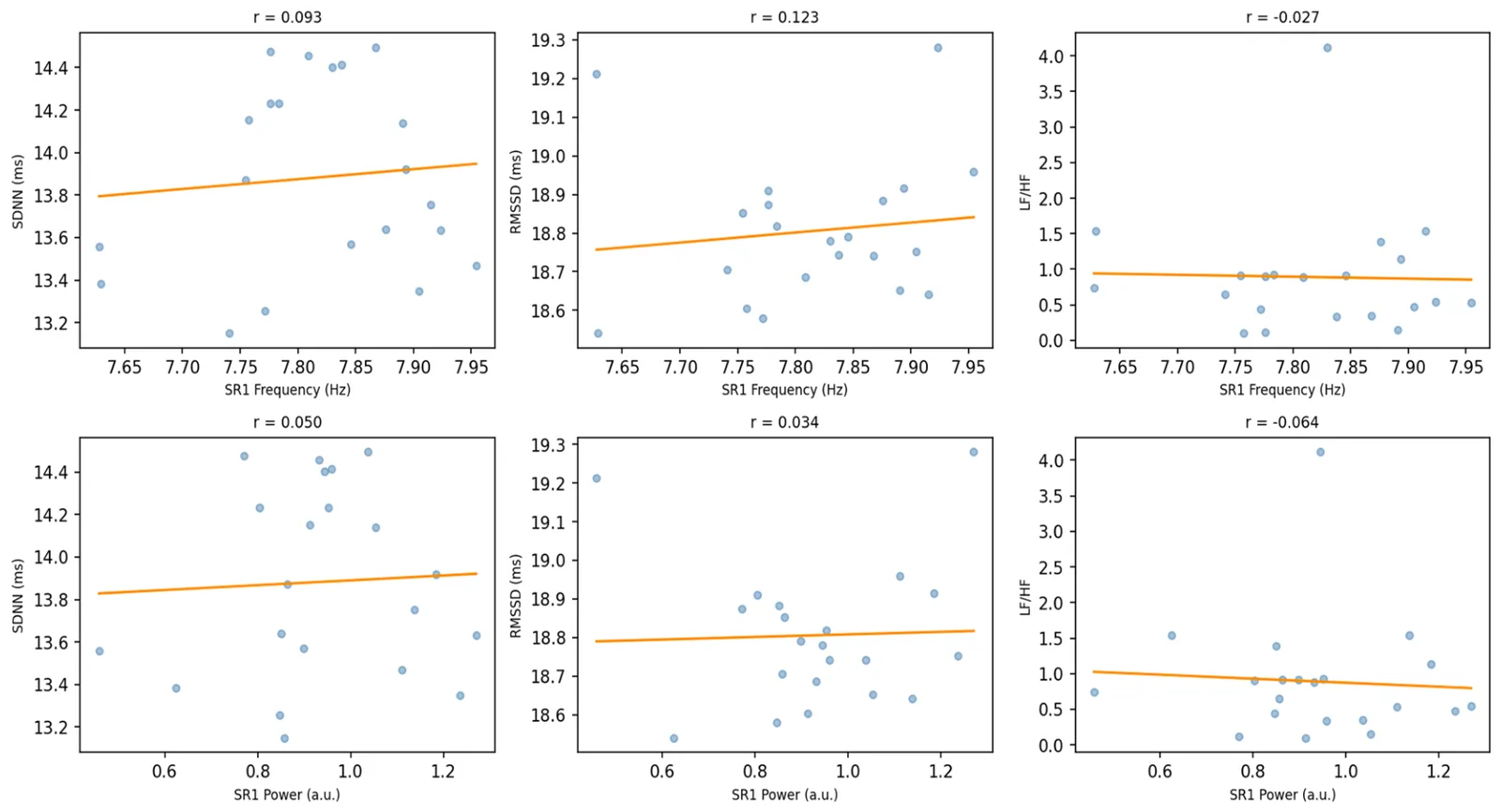
Partial correlation scatter plots (simulation): raw HRV–SR correlation (upper panel) vs. Kp-adjusted residual correlation (lower panel).

Part B: Pipeline Validation and Simulation (Section 3.4, Section 3.5 and Section 3.6). Section 3.4 uses real PhysioNet ECG data for pipeline validation (Figure 4, Figure 5 and Figure 6); Section 3.5 and Section 3.6 use simulated data (Figure 7, Figure 8, Figure 9 and Figure 10). No SR–HRV physiological inference is made in this part.

Record 55 (AF-dominant) shows highly irregular RRI (300–1800 ms range); Records 00 and 08 show normal nocturnal heart rate reduction. These records are used solely to validate HRV feature extraction and are not synchronized with any SR or geomagnetic data.

Record 55 (AF) shows markedly elevated values (SDNN ~84 ms, RMSSD ~110 ms). HRV indices from AF segments (Record 55) do not reflect autonomic modulation; they confirm that the pipeline handles irregular RRI without error.

LF/HF is highest in Record 08; note that the LF/HF ratio does not constitute a direct or universal index of sympathoadrenal balance, particularly in long-term ambulatory recordings, and no interpretation of sympathetic dominance is intended here. Spectral HRV indices from AF segments (Record 55) do not reflect autonomic tone and are presented for pipeline completeness only.

The SR modal structure (SR1–SR4) is reproduced at 7.83, 14.3, 20.8, and 27.3 Hz. This simulated PSD illustrates the expected resonance morphology and provides the baseline for subsequent simulations of SR frequency variability (Figure 8) and HRV–SR–Kp interactions (Figure 9).

Red bands indicate simulated geomagnetic storm events; gray dotted lines show nominal SR frequencies (7.83, 14.3, 20.8, 27.3 Hz). Diurnal variation is driven by ionospheric day–night conductivity changes (terminator effect), not by human circadian rhythm.

The simulation also explored scenarios where broadband voltage SD changes and HRV changes do not coincide with Kp elevations, illustrating that a future empirical study may observe residual SR–HRV co-variation independent of geomagnetic confounding. Whether such residual co-variation exists in real synchronized data is the central empirical question that this pipeline is designed to test.

Removing Kp-driven variance reduces the apparent HRV–SR correlation (Δr ≈ 0.07–0.12), confirming that geomagnetic activity acts as a confound. Results are from simulated data and illustrate pipeline behavior only.

### 3.4. HRV Pipeline Validation (PhysioNet LTAFDB)

Prior to applying the HRV analysis pipeline to the ALLSTAR database, its correctness was verified using three PhysioNet LTAFDB records (Section 2.8). Figure 4, Figure 5 and Figure 6 show the HRV time series extracted from these records. As these records include an AF case (Record 55), HRV indices derived from AF segments are not physiologically interpretable but confirm pipeline robustness across diverse RRI dynamics. These figures are entirely independent of the SR and geomagnetic datasets.

### 3.5. Conceptual Three-Way Framework: HRV × SR × Kp (Simulation-Based Illustration)

To introduce the simulation framework, we first reproduced the expected Schumann resonance modal structure using a simulated ELF waveform. Figure 7 illustrates the Welch PSD of a representative 35-min segment, showing clear SR1–SR4 peaks. This PSD serves as the foundation for subsequent simulations of SR frequency variability (Figure 8) and HRV–SR–Kp interactions (Figure 9).

### 3.6. Simulation-Based Validation of the Partial Correlation Framework

The partial correlation framework was validated using simulated data generated from published summary statistics (see Section 2.7). Representative simulation results are as follows. In the absence of Kp control, a positive raw association was observed between SR1 frequency and SDNN (r_raw = 0.28) and between SR1 power and RMSSD (r_raw = 0.21). These values were chosen to be representative of effect sizes reported in the prior literature on geophysical–physiological associations [18,19]. After partial correlation controlling for Kp, both associations were attenuated (r_partial(SR1 frequency, SDNN) = 0.19; r_partial(SR1 power, RMSSD) = 0.14; Δr ≈ 0.07–0.09). Critically, these figures are derived from a simulation, not from paired observations of SR and HRV measured simultaneously. They should therefore be interpreted only as validation of the pipeline’s ability to detect and quantify geomagnetic confounding, not as evidence of an empirical SR–HRV association.

For the simulation of n = 1000 paired observations, the raw correlations r_raw = 0.28 and r_raw = 0.21 were statistically significant (both *p* < 0.001; 95% CI: 0.23–0.33 and 0.15–0.27, respectively). After Kp adjustment, the partial correlations r_partial = 0.19 and r_partial = 0.14 remained statistically significant (p < 0.001). Because these statistics are derived from pre-specified simulation parameters, significance reflects computational accuracy of the pipeline, not any empirical finding about SR–HRV coupling.

The simulation demonstrates that Kp-driven confounding can account for a non-trivial fraction (Δr ≈ 0.07–0.12) of the apparent SR–HRV association, validating the necessity of including the Kp partial correlation step in any future empirical analysis. The LF/HF ratio showed a small simulated negative partial correlation with SR1 frequency (r_partial ≈ −0.12), consistent with the hypothesis that lower SR fundamental frequency during geomagnetic disturbances may co-occur with sympathetic dominance; however, this remains speculative until tested with synchronized data.

### 3.7. Feasibility of Integrating Environmental Electromagnetic Data with Large-Scale HRV Data

Although the SR dataset and the ALLSTAR Holter ECG recordings were collected at different locations and time periods, the present analyses demonstrate the feasibility of combining environmental electromagnetic monitoring data with large physiological datasets. The observed variability in broadband voltage SD within short time windows, together with the large population-level variability in HRV spectral indices, demonstrates that both data types exhibit sufficient variability to be detectable in future synchronized analyses, satisfying a necessary (though not sufficient) condition for testing the SR-HRV hypothesis. No physiological inference about SR-HRV interaction is drawn from this comparison of non-synchronized datasets.

These results provide a methodological foundation for future studies involving simultaneous measurements of Schumann resonance and multi-day Holter ECG recordings, which may enable direct investigation of temporal associations between global electromagnetic activity and cardiovascular autonomic dynamics.

Table 1 lists summary statistics for all measurement sessions. N = 525,000 samples per segment (250 Hz × 35 min).

The measurements on April 4th and May 5th had a larger SD (standard deviation) than the other dates, indicating greater variability within the data.

“Average” represents the temporal mean of AC-coupled voltage (near zero by design; values reflect residual DC offset, not signal amplitude). “SD” is the standard deviation of the voltage time series and reflects broadband signal variability (higher SD = greater within-segment fluctuation; this does not equate to calibrated SR power). Sessions on 4 April and 5 May 2022 show markedly larger SD values, coinciding with elevated Kp geomagnetic activity on those dates.

Note: The segment-wise mean voltage reflects residual DC offset after AC coupling and does not represent physical SR amplitude.

## 4. Discussion

The present study explored the feasibility of integrating large-scale physiological datasets with environmental electromagnetic monitoring to investigate potential relationships between Schumann resonance (SR) variability and human autonomic cardiovascular dynamics. Using publicly available SR data and a large ambulatory electrocardiographic database, we examined the statistical characteristics of broadband voltage SD and population-scale HRV spectral indices. The results demonstrated that broadband voltage SD exhibits substantial variability over relatively short time scales, and that large-scale physiological databases provide a promising framework for exploring environmental influences on autonomic regulation.

One notable finding of this study is the substantial variability observed in broadband voltage SD within relatively short measurement intervals. Analysis of the Mendeley Data SR dataset showed that the standard deviation of the AC-coupled voltage within individual 35-min measurement windows was relatively large, indicating that broadband signal variability is considerable even over short periods. Furthermore, the mean voltage level also differed across measurement sessions within the same day, suggesting that broadband signal variability shows significant intra-day variation. These findings highlight the dynamic nature of the global electromagnetic environment and suggest that SR signals may reflect continuously changing atmospheric and ionospheric conditions.

Prior theoretical work has proposed that SR frequencies (7.83 Hz and harmonics) overlap with the extremely low frequency (ELF) band and with components of biological rhythms, suggesting the possibility of environmental electromagnetic influences on physiological regulation [6,7,8]. However, empirical evidence from controlled studies remains limited and contested, and the present study did not measure EEG or any neurophysiological signal. The Discussion section therefore does not pursue mechanistic biological speculation; instead, it focuses on the methodological implications of the analysis pipeline for designing future empirical tests of this hypothesis.

The principal contribution of the present study is methodological rather than empirical. The most important design constraint—the absence of temporal synchronization between the SR dataset (Trinidad and Tobago, 2022) and the Holter ECG database (Japan, multiple years)—means that observed statistical summaries cannot be interpreted as physiological associations between SR and HRV in the same population or time window. This limitation is not a flaw in the analysis; it reflects the current state of open-access data availability. The study contribution is to characterize each data source, propose an integrated pipeline, and identify what a future synchronized dataset must look like in order to enable definitive empirical testing.

The absence of temporally and spatially synchronized data is the central limiting factor of this study and substantially restricts the conclusions that can be drawn. The Schumann resonance recordings (Trinidad and Tobago, 2022), the geomagnetic Kp index (NOAA global), and the ambulatory ECG data (ALLSTAR, Japan, multiple years) were collected from different geographic locations, during different time periods, and in different populations. Consequently, these datasets cannot be used to assess actual empirical associations between Schumann resonance activity and heart rate variability. No wording in this manuscript is intended to suggest that an empirical environmental–physiological association has been demonstrated.

To partially mitigate the absence of temporal synchronization, the present study characterized each dataset at the population level: HRV spectral indices summarized across ~730,000 recordings, and broadband voltage SD statistics summarized across ~525,000 samples per session. Population-level summary statistics are informative for pipeline design even when individual-level synchronization is unavailable because they establish the expected variability ranges that any future synchronized study would need to resolve. However, it must be emphasized that population-level comparison of two unrelated datasets does not constitute evidence of a physiological association, and no such claim is made here.

A methodological contribution of the present study is the explicit treatment of the NOAA Kp geomagnetic index as a confounding variable and the introduction of a partial correlation framework to control for it. Geomagnetic activity can independently modulate both SR spectral characteristics [5] and autonomic cardiovascular indices [18], so raw SR–HRV correlations in any future dataset may reflect shared geomagnetic forcing rather than direct SR–HRV coupling. Simulation results show that Kp-driven confounding can account for Δr ≈ 0.07–0.12 of the apparent association, depending on the index pair. Future studies reporting SR–HRV associations should routinely present both raw and Kp-adjusted correlation coefficients to allow proper interpretation and cross-study comparability.

Nevertheless, several limitations should be considered when interpreting the results. The most important limitation is the lack of precise time information in the SR dataset. Because the exact measurement times were not recorded, it was not possible to examine simultaneous fluctuations between SR signals and HRV indices. As a result, the present study could only evaluate statistical characteristics of SR variability and the feasibility of combining environmental and physiological datasets. Future studies will require datasets with precisely recorded timestamps in order to perform rigorous time-series analyses of environmental–physiology interactions.

The following additional methodological limitations are also acknowledged:

- The broadband voltage SD in the SR dataset does not represent SR power; it is used only as a descriptive statistic of segment variability.
- The simulation is illustrative and parameter-dependent, intended solely to validate the analysis pipeline.

Precise recording timestamps are absent from the SR dataset; the date-level Kp context (Table S2) reflects verified NOAA archival data, but the sub-daily alignment of 3-h Kp blocks to individual SR windows is an approximation and should be treated as an illustrative temporal covariate, not a precisely determined session-level measurement.

- HRV indices from AF segments (LTAFDB) are not physiologically interpreted; ALLSTAR HRV statistics are derived from normal sinus rhythm only.

Another limitation concerns the spatial separation between the SR monitoring site and the physiological dataset. The SR measurements analyzed in this study were recorded at a monitoring site in Trinidad and Tobago, whereas the Holter ECG recordings in the ALLSTAR database were collected across Japan. Although SR signals reflect global electromagnetic processes, regional differences in electromagnetic environments may still influence the observed signals. Therefore, simultaneous measurements conducted within the same geographic region would further strengthen future analyses.

Future research could address these limitations by integrating physiological datasets with environmental monitoring systems that provide precise time information. For example, publicly available physiological waveform databases such as the MIT-BIH Normal Sinus Rhythm Database and the MIMIC-III Waveform Database available through PhysioNet provide ECG recordings with well-defined timestamps. Combining such datasets with continuous SR monitoring data could enable direct time-series analyses of potential interactions between environmental electromagnetic variability and human autonomic regulation.

Another promising direction is the use of long-term ambulatory ECG monitoring together with simultaneous environmental monitoring. For example, multi-day Holter ECG recordings (e.g., 7-day monitoring) combined with continuous SR observations could allow direct evaluation of temporal associations between SR fluctuations and HRV dynamics. Such studies may be particularly informative during periods of enhanced geomagnetic activity, such as the current solar cycle maximum.

A major methodological constraint is the geographic mismatch between the SR dataset and the physiological data. SR measurements from Trinidad and Tobago (10.7 deg N, 61.1 deg W) are not directly representative of SR conditions in Japan, where the ALLSTAR database was collected. Although SR is a global resonance phenomenon, the instantaneous SR amplitude and frequency at any observation point depend on the observer position relative to active thunderstorm regions and on cavity propagation geometry [26]. Future synchronized studies must therefore collect SR data at or near the geographic location of the physiological monitoring and should account for local electromagnetic background and power-grid interference.

The temporal scope of the SR dataset is a further limitation. The Mendeley SR dataset covers only six observation days spanning February to May 2022, representing a single season during the ascending phase of Solar Cycle 25. SR characteristics vary substantially with season (driven by latitudinal shifts in global lightning activity) and with solar cycle phase (due to ionospheric conductivity changes). The six-day sample cannot provide a representative estimate of SR variability across seasons or solar cycle phases, and the simulation parameters derived from it have correspondingly limited external validity. Future synchronized studies should span multiple seasons and should include solar cycle phase as an additional covariate.

The simulation presented here demonstrates the operation of the Kp partial correlation framework but does not constitute full methodological validation. The simulated delta-r values (0.07–0.12) depend on assumed correlation structures parameterized from non-synchronized datasets. A rigorous Monte Carlo sensitivity analysis across multiple scenarios—including no true SR-HRV association, Kp as a common cause, Kp-independent SR-HRV association, lagged effects, non-linear effects, serial autocorrelation, measurement error, and multiple correlated environmental predictors—should be conducted and reported in any future empirical study that applies this pipeline to synchronized data.

The appropriate causal role of Kp requires explicit pre-specification. The present analysis treats Kp as a nuisance confounder to be partialled out; however, Kp may instead represent an upstream exposure indicator, a component of the environmental pathway of interest, or an imperfect proxy for broader geomagnetic conditions. Future empirical studies should specify a directed acyclic graph (DAG) that defines the causal role of Kp and other environmental covariates relative to SR and HRV, rather than assuming a priori that Kp is always a confounder. Additional covariates—atmospheric temperature, barometric pressure, and local EMF noise—should also be considered in the analysis model.

The present manuscript clearly distinguishes conceptual claims (what the pipeline is designed to test) from empirically demonstrated findings (which are limited to simulation outputs). A directed acyclic graph (DAG) formalizing the assumed causal structure—with Kp as a common cause of both SR variability and HRV, and with SR as a potential additional direct cause of HRV—should be pre-specified and reported in any future empirical study. The current analysis treats Kp as a nuisance confounder; alternative causal structures (Kp as mediator, Kp as proxy for unmeasured ionospheric state) should be explored using sensitivity analysis or instrumental variable methods in future work.

A fundamental design challenge for future synchronized studies is the temporal resolution mismatch between HRV and SR metrics. ALLSTAR HRV indices represent 24-h aggregate measures, whereas SR data consist of 35-min segments. Meaningful statistical association requires matched temporal windows. Future studies should compute short-window HRV indices (e.g., 5-min spectral HRV following Task Force guidelines) from ambulatory ECG recordings with precise GPS-referenced timestamps, aligned to SR measurement windows of matching duration. Multiple window lengths and lag structures should be explored, as the time scale of any putative SR-biological interaction remains unknown.

Overall, the present study demonstrates the feasibility of combining global environmental electromagnetic data with large-scale physiological datasets. The substantial variability observed in broadband voltage SD within short time intervals highlights the dynamic nature of the electromagnetic environment surrounding the Earth. At the same time, the availability of extremely large HRV datasets, such as the ALLSTAR dataset, provides the statistical power needed to detect small environmental effects if they exist. The explicit treatment of the Kp geomagnetic index as a confounding variable and the introduction of a partial correlation framework are methodological advances that any future empirical study in this domain should adopt. These results provide a methodological foundation for future studies involving simultaneous measurements of Schumann resonance, geomagnetic activity, and multi-day Holter ECG recordings. When such synchronized datasets become available, the proposed pipeline will enable direct investigation of temporal associations between global electromagnetic dynamics and cardiovascular autonomic regulation, while rigorously separating true SR–HRV coupling from geomagnetic confounding. The present feasibility study therefore defines both the analytical requirements and the data-collection specifications necessary for a definitive empirical evaluation of environmental electromagnetic influences on human autonomic physiology.

## 5. Conclusions

This study presents a methodological feasibility assessment for integrating three complementary open-access data sources—the ALLSTAR Holter ECG database, the Mendeley Schumann Resonance Dataset, and the NOAA Kp geomagnetic index—into a unified analysis pipeline for future SR–HRV research. Because the available datasets were not collected at the same time or location, the study makes no empirical claim about a physiological association between SR dynamics and human autonomic cardiovascular regulation. All reported association statistics are simulation-based illustrations of the pipeline, not empirical measurements.

Independent characterization of each data source revealed the following: (1) substantial within-session and cross-day variability in broadband voltage SD (SD range 2.4–5.2 across observation days), indicating that SR monitoring requires high-cadence time-stamped recordings; (2) large population-level variability in HRV spectral indices across ~730,000 recordings, providing the statistical power needed to detect small environmental effects if they exist; and (3) Kp activity levels ranging from quiet to moderate storm conditions during the SR observation.

The central methodological contribution is a partial correlation framework that removes Kp-driven variance from both SR features and HRV indices before computing their association, evaluated through simulation (Δr ≈ 0.07–0.12). Future studies that obtain time-synchronized SR and Holter ECG recordings—ideally multi-day recordings with GPS-timestamped SR data from a monitoring station in the same geographic region—should apply this pipeline to test whether a residual SR–HRV association survives Kp adjustment. The present study is not evidence for or against such an association; it is a methodological template and feasibility demonstration that defines what the next empirical study must do.

## Figures and Tables

**Table 1:** Measurement data list (all items).

Data	Name	Average	SD	N
02/20	R1 ~ R4	−0.0130	2.7639	525,000
03/02	R5 ~ R6	−0.0683	2.4188	525,000
03/17	R7 ~ R11	−0.0353	3.4042	525,000
04/04	R12 ~ R15	−0.0064	4.8173	525,000
05/01	R16 ~ R28	−0.0718	3.6510	525,000
05/05	R29 ~ R38	−0.0163	5.2456	525,000
05/11	R39 ~ R50	−0.0210	3.4402	525,000

## Data Availability

The data supporting the findings of this study are available from the following publicly accessible repositories: ALLSTAR (Allostatic State Mapping by Ambulatory ECG Repository): https://allstar.jpn.org/ (accessed on 22 July 2026). NOAA Kp and ap Index: https://www.ncei.noaa.gov/products/geomagnetic-indices. Schumann’s Resonance Data Set: Available via Mendeley Data. The dataset titled “Number vs. voltage recording data set for Schumann’s resonance analysis at 250 samples per second” is available via Mendeley Data. It was published on 5 July 2022, by Oba Ranu. The data were collected using a portable coil antenna and detection circuitry, sampling Schumann resonance voltage signals at 250 Hz. Measurements were conducted in a low-noise environment in northern Trinidad, away from the 60 Hz power grid. The dataset can be accessed at the following DOI: https://doi.org/10.17632/mzkx83bnr9.1.

## Supplementary Materials

The following supporting information can be downloaded at: https://www.mdpi.com/article/10.3390/s26216742/s1. Table S1: ALLSTAR database dataset—HRV indicators (SDNN, RMSSD, pNN50, LF/HF ratio) calculated by FFT from 738,464 twenty-four-hour Holter ECG recordings. This dataset is governed by the data access and anonymization policies of the ALLSTAR project and is not provided as a direct download attachment. Qualified researchers may request access through the ALLSTAR project website https://allstar.jpn.org/ (accessed on 22 July 2026). Population-level summary statistics are presented in Figure 2 of the manuscript. Table S2: Session-level SR recording metadata (recording identifier, date, local recording window, SD of AC-coupled voltage, mean Kp, and geomagnetic condition) for all seven observation sessions in the Mendeley Schumann Resonance Dataset (February–May 2022). Code S1: Complete annotated Python simulation script for the SR–HRV partial correlation pipeline validation. The script implements three Monte Carlo scenarios (n = 1000 iterations each) to quantify Kp-confounding effects on raw and partial SR–HRV correlations (Python 3.10.7; NumPy 1.23.5, SciPy 1.9.3, Matplotlib 3.6.2; random seed = 42).

## Abbreviations

The following abbreviations are used in this manuscript:

SR	Schumann Resonance
HRV	Heart Rate Variability
ECG	Electrocardiogram
RR interval (RRI)	Interval Between Successive R-waves in the ECG Signal
FFT	Fast Fourier Transform
PSD	Power Spectral Density
SD	Standard Deviation
ELF	Extremely Low Frequency
EEG	Electroencephalography
ALLSTAR	Allostatic State Mapping by Ambulatory ECG Repository
LF	Low-Frequency Component of HRV
HF	High-Frequency Component of HRV
SDNN	Standard Deviation of Normal-to-Normal Intervals
PhysioNet	Physiological Signal Database Repository
SR dataset	Schumann Resonance Dataset (Mendeley Data)
