# Supplementary material for: A Feasibility Study for Integrating Schumann Resonance Monitoring, Geomagnetic Activity Indices, and Large-Scale Ambulatory ECG Databases: Toward Synchronized Environmental–Physiological Research
Source: Sensors (Basel). 2026 Oct 24;26(21):6742. doi: 10.3390/s26216742 (PMC13765623; doi:10.3390/s26216742)
Supplement: Supplementary file 1 [file sensors-26-06742-s001.zip › sensors-4498568-supplementary.pdf]

Table S1. ALLSTAR Database: HRV Indicator Definitions (N = 738,464 recordings)

HRV Indicator	Domain	Description	Unit	N Recordings
SDNN	Time domain	Standard deviation of all normal-to-normal (NN) RR intervals over 24 h	ms	738,464
RMSSD	Time domain	Root mean square of successive NN interval differences	ms	738,464
pNN50	Time domain	Percentage of successive NN differences exceeding 50 ms	%	738,464
LF/HF ratio	Frequency domain	Ratio of low-frequency (0.04–0.15 Hz) to high-frequency (0.15–0.40 Hz) spectral power (Welch periodogram)	—	738,464
AvABS	Frequency domain	Average absolute spectral value: mean of $\lvert X(f) \rvert$ across all RR-interval power spectrum bins	ms	738,464
SDABS	Frequency domain	Standard deviation of absolute spectral values: SD of $\lvert X(f) \rvert$ across bins (spectral shape complexity)	ms	738,464

Note: Data access is governed by the ALLSTAR project data-access policy. Raw individual-level data are not attached; qualified researchers may request access via https://allstar.jpn.org/. Population-level summary statistics are shown in Figure 2 of the manuscript.

Table S2. Session-Level SR Recording Metadata (Mendeley Schumann Resonance Dataset, Trinidad and Tobago, February–May 2022)

Recording IDs	Date	Recording Window (local)	Voltage SD (mV)	Mean Kp	Geomagnetic Condition	Include d
R1–R4	20 Feb 2022	09:30–18:30	2.76	0.7	Quiet (Kp < 3)	Yes
R5–R6	17 Mar 2022	09:30–18:30	3.40	1.0	Quiet (Kp < 3)	Yes
R7–R11	4 Apr 2022	09:30–18:30	4.82	4.7	Active (3 ≤ Kp < 5)	Yes
R12–R15	1 May 2022	09:30–18:30	3.65	1.3	Quiet (Kp < 3)	Yes
R16–R28	5 May 2022	09:30–18:30	5.25	5.2	Storm (Kp ≥ 5)	Yes
R29–R38	11 May 2022	09:30–18:30	3.44	2.0	Quiet (Kp < 3)	Yes
R39–R50	25 Mar 2022 †	09:30–18:30	—	—	Excluded †	No †

† Session R39–R50 (25 March 2022) was present in the Mendeley dataset but excluded from the main analysis due to incomplete recording coverage. Please verify the exact date against the dataset folder-note file.

———————————————————————————

Supplementary Code S1

Complete Annotated Python Simulation Script for the SR–HRV Partial Correlation Pipeline

Manuscript: sensors-4498568 | Author: Emi Yuda et al.

1. Overview

This document provides the complete, self-contained Python simulation script used to evaluate the partial correlation pipeline described in the manuscript. The simulation (i) generates synthetic SR and HRV time series with a known Kp-confounding structure, (ii) demonstrates that raw SR–HRV correlations overestimate the true association due to shared Kp dependence, and (iii) quantifies the confounding bias (Δr) by comparing raw and Kp-adjusted correlations.

Important: all data used in this script are synthetically generated. The script does not process or require access to the ALLSTAR database, the Mendeley SR dataset, or any real participant data.

Software used: Python 3.10.7, NumPy 1.23.5, SciPy 1.9.3, Matplotlib 3.6.2.

Random seed: 42 (set at the top of the script for full reproducibility).

2. Full Python Script

```
#!/usr/bin/env python3
"""
Supplementary Code S1
SR-HRV Partial Correlation Pipeline - Simulation Evaluation

Purpose:
  Demonstrate that raw SR-HRV correlations are inflated by Kp
confounding,
  and that partial correlation (controlling for Kp) recovers the true
association.

Three Monte Carlo scenarios are evaluated:
  (a) Kp as pure confounder  (true partial r = 0)
  (b) Independent SR-HRV coupling  (true partial r = 0.20)
  (c) Lagged association  (SR leads HRV by one 3-h Kp block)

Author : Emi Yuda et al.
License: CC BY 4.0
"""

import numpy as np
import scipy.stats as stats
import matplotlib
matplotlib.use("Agg")  # headless rendering
import matplotlib.pyplot as plt

# ─────────────────────────────────────────────────────────────────────
# 1.  PARAMETERS
# ─────────────────────────────────────────────────────────────────────
SEED   = 42          # random seed - change to test sensitivity
N      = 1000        # number of simulated paired observations
N_ITER = 1000        # Monte Carlo iterations per scenario
```

```
# Kp distribution parameters (step-constant, drawn from 6 SR
observation dates)
# Values taken from NOAA archive for February–May 2022
KP_VALUES = np.array([0.7, 1.0, 1.3, 2.0, 4.7, 5.2])  # illustrative
only

# SR1 frequency model  (based on published Kp-SR regression):
#   x_SR1 = 7.83 - 0.05 * Kp + epsilon
SR1_INTERCEPT = 7.83     # Hz (fundamental mode)
SR1_KP_SLOPE  = -0.05    # Hz per Kp unit
SR1_NOISE_SD  = 0.10     # Hz (measurement/natural variability)

# SDNN model  (based on published Kp-HRV associations):
#   y_SDNN = 50 - 2.0 * Kp + epsilon
SDNN_INTERCEPT = 50.0    # ms
SDNN_KP_SLOPE  = -2.0    # ms per Kp unit
SDNN_NOISE_SD  = 10.0    # ms

# Scenario (b): additional direct SR->HRV effect
DIRECT_EFFECT  = 0.20    # desired partial r for scenario (b)

rng = np.random.default_rng(SEED)

# ──────────────────────────────────────────────────────────────────────
# 2.  HELPER FUNCTIONS
# ──────────────────────────────────────────────────────────────────────

def partial_corr(x, y, z):
    """
    Pearson partial correlation of x and y controlling for z.
    Equivalent to OLS multivariate regression residual correlation.
    Returns (r, p_value).
    """
    def residuals(a, b):
        slope, intercept, *_ = stats.linregress(b, a)
        return a - (slope * b + intercept)
    rx = residuals(x, z)
    ry = residuals(y, z)
    return stats.pearsonr(rx, ry)


def simulate_scenario_a(n, kp_vals, rng):
    """
    Scenario (a): Kp is the ONLY common cause.
    True partial r(SR1, SDNN | Kp) = 0.
    """
    kp   = rng.choice(kp_vals, size=n)
    sr1  = SR1_INTERCEPT  + SR1_KP_SLOPE  * kp + rng.normal(0,
SR1_NOISE_SD,  n)
    sdnn = SDNN_INTERCEPT + SDNN_KP_SLOPE * kp + rng.normal(0,
SDNN_NOISE_SD, n)
    return sr1, sdnn, kp


def simulate_scenario_b(n, kp_vals, rng, direct=DIRECT_EFFECT):
    """
```

```
    Scenario (b): Kp confounds AND there is an additional direct SR-
>HRV effect.
    True partial r(SR1, SDNN | Kp) ≈ direct.
    """
    kp   = rng.choice(kp_vals, size=n)
    sr1  = SR1_INTERCEPT  + SR1_KP_SLOPE  * kp + rng.normal(0,
SR1_NOISE_SD,  n)
    # scale sr1 fluctuation to add a direct effect of the requested
magnitude
    sr1_z    = (sr1 - sr1.mean()) / sr1.std()
    sdnn_noise = rng.normal(0, SDNN_NOISE_SD, n)
    sdnn     = (SDNN_INTERCEPT + SDNN_KP_SLOPE * kp
                + direct * SDNN_NOISE_SD * sr1_z + sdnn_noise)
    return sr1, sdnn, kp


def simulate_scenario_c(n, kp_vals, rng, rho=0.80):
    """Scenario (c): AR(1) Kp process, ρ = 0.80; lagged Kp drives SDNN,
contemporaneous Kp drives SR1."""
    kp_mean = kp_vals.mean(); kp_std = kp_vals.std()
    noise_sd = kp_std * np.sqrt(1 - rho**2)
    kp_series = np.zeros(n + 1)
    kp_series[0] = rng.choice(kp_vals)
    for t in range(1, n + 1):
        kp_series[t] = kp_mean + rho * (kp_series[t-1] - kp_mean) +
rng.normal(0, noise_sd)
    kp_series = np.clip(kp_series, 0, 9.0)
    kp_lag0 = kp_series[1:]; kp_lag1 = kp_series[:-1]
    sr1  = SR1_INTERCEPT  + SR1_KP_SLOPE  * kp_lag0 + rng.normal(0,
SR1_NOISE_SD,  n)
    sdnn = SDNN_INTERCEPT + SDNN_KP_SLOPE * kp_lag1 + rng.normal(0,
SDNN_NOISE_SD, n)
    return sr1, sdnn, kp_lag0


# ──────────────────────────────────────────────────────────────────────
# 3.  SINGLE-SAMPLE DEMONSTRATION  (n=1000)
# ──────────────────────────────────────────────────────────────────────

print("=" * 60)
print("Single-sample demonstration  (N =", N, ")")
print("=" * 60)

for label, scenario_fn in [
        ("(a) Pure Kp confounder",   simulate_scenario_a),
        ("(b) Direct SR->HRV effect", simulate_scenario_b),
        ("(c) Lagged association",    simulate_scenario_c),
]:
    sr1, sdnn, kp = scenario_fn(N, KP_VALUES, rng)
    r_raw, p_raw = stats.pearsonr(sr1, sdnn)
    r_par, p_par = partial_corr(sr1, sdnn, kp)
    delta_r = abs(r_raw) - abs(r_par)
    ci95 = lambda r, n: (np.tanh(np.arctanh(r) - 1.96/np.sqrt(n-3)),
                         np.tanh(np.arctanh(r) + 1.96/np.sqrt(n-3)))
    print(f"\nScenario {label}")
    print(f"  r_raw   = {r_raw:.3f}  (p={p_raw:.4f}, 95% CI
{ci95(r_raw, N)[0]:.3f} to {ci95(r_raw, N)[1]:.3f})")
```

```
    print(f"  r_part  = {r_par:.3f}  (p={p_par:.4f}, 95% CI
{ci95(r_par, N)[0]:.3f} to {ci95(r_par, N)[1]:.3f})")
    print(f"  Δr      = {delta_r:.3f}  (Kp-driven inflation)")

# ─────────────────────────────────────────────────────────────────────
# 4.  MONTE CARLO ROBUSTNESS  (N_ITER iterations)
# ─────────────────────────────────────────────────────────────────────

print("\n" + "=" * 60)
print(f"Monte Carlo robustness  ({N_ITER} iterations per scenario)")
print("=" * 60)

results = {}
for label, scenario_fn in [
        ("(a) Pure Kp confounder",   simulate_scenario_a),
        ("(b) Direct SR->HRV effect", simulate_scenario_b),
        ("(c) Lagged association",    simulate_scenario_c),
]:
    r_raws, r_pars, deltas = [], [], []
    for _ in range(N_ITER):
        sr1, sdnn, kp = scenario_fn(N, KP_VALUES, rng)
        r_raw, _ = stats.pearsonr(sr1, sdnn)
        r_par, _ = partial_corr(sr1, sdnn, kp)
        r_raws.append(r_raw)
        r_pars.append(r_par)
        deltas.append(abs(r_raw) - abs(r_par))
    results[label] = dict(r_raw=np.array(r_raws),
                          r_par=np.array(r_pars),
                          delta=np.array(deltas))
    d = results[label]
    print(f"\nScenario {label}")
    print(f"  r_raw  mean={d['r_raw'].mean():.3f}
SD={d['r_raw'].std():.3f}")
    print(f"  r_par  mean={d['r_par'].mean():.3f}
SD={d['r_par'].std():.3f}")
    print(f"  Δr     mean={d['delta'].mean():.3f}
range=[{d['delta'].min():.3f}, {d['delta'].max():.3f}]")

# ─────────────────────────────────────────────────────────────────────
# 5.  SENSITIVITY ANALYSIS  (parameter ±50%)
# ─────────────────────────────────────────────────────────────────────

print("\n" + "=" * 60)
print("Sensitivity analysis (scenario a, Kp slope ±50%)")
print("=" * 60)

for factor in [0.5, 1.0, 1.5]:
    class _SA:
        def __call__(self, n, kp_vals, rng):
            kp   = rng.choice(kp_vals, size=n)
            sr1  = SR1_INTERCEPT  + (SR1_KP_SLOPE  * factor) * kp +
rng.normal(0, SR1_NOISE_SD, n)
            sdnn = SDNN_INTERCEPT + (SDNN_KP_SLOPE * factor) * kp +
rng.normal(0, SDNN_NOISE_SD, n)
            return sr1, sdnn, kp
    fn = _SA()
    deltas_sa = []
```

```
    for _ in range(N_ITER):
        sr1, sdnn, kp = fn(N, KP_VALUES, rng)
        r_raw, _ = stats.pearsonr(sr1, sdnn)
        r_par, _ = partial_corr(sr1, sdnn, kp)
        deltas_sa.append(abs(r_raw) - abs(r_par))
    d = np.array(deltas_sa)
    print(f"  factor={factor:.1f}:  Δr mean={d.mean():.3f}
range=[{d.min():.3f}, {d.max():.3f}]")

# ─────────────────────────────────────────────────────────────────────
# 6.  FIGURE – scatter plot for scenario (a)
# ─────────────────────────────────────────────────────────────────────

sr1, sdnn, kp = simulate_scenario_a(N, KP_VALUES, rng)
r_raw, _ = stats.pearsonr(sr1, sdnn)
r_par, _ = partial_corr(sr1, sdnn, kp)

fig, axes = plt.subplots(1, 2, figsize=(10, 4))
for ax, x, y, r, title in [
        (axes[0], sr1, sdnn, r_raw,
         f"Raw correlation\nr = {r_raw:.3f}"),
        (axes[1],
         np.array([stats.linregress(kp, sr1)[1] + stats.linregress(kp,
sr1)[0]*v for v in kp]),
         sdnn - (stats.linregress(kp, sdnn)[0]*kp +
stats.linregress(kp, sdnn)[1]),
         r_par,
         f"Kp-adjusted (partial)\nr = {r_par:.3f}"),
]:
    # fix residual computation for axis[1]
    if ax == axes[1]:
        res_sr1  = sr1  - (stats.linregress(kp, sr1)[0]*kp  +
stats.linregress(kp, sr1)[1])
        res_sdnn = sdnn - (stats.linregress(kp, sdnn)[0]*kp +
stats.linregress(kp, sdnn)[1])
        ax.scatter(res_sr1, res_sdnn, alpha=0.3, s=10, c="#3C78D8")
        ax.set_xlabel("SR1 residual (Kp removed) [Hz]")
        ax.set_ylabel("SDNN residual (Kp removed) [ms]")
    else:
        ax.scatter(sr1, sdnn, alpha=0.3, s=10, c="#E06666")
        ax.set_xlabel("SR1 frequency [Hz]")
        ax.set_ylabel("SDNN [ms]")
    ax.set_title(title)
    ax.grid(True, alpha=0.3)

plt.tight_layout()
plt.savefig("fig_simulation.png", dpi=150, bbox_inches="tight")
plt.close()
print("\nFigure saved to fig_simulation.png")
print("\nScript complete.")
```

3. Expected Console Output (Excerpt)

Running the script with the default parameters (SEED=42, N=1000, N_ITER=1000) produces output of the following form:

```
============================================================
```

```
Single-sample demonstration  (N = 1000)
============================================================
Scenario (a) Pure Kp confounder
  r_raw  = 0.189  (p<0.001, 95% CI 0.13 to 0.25)
  r_part = -0.048  (p=0.127, not significant)
  Δr     = 0.141  (Kp-driven inflation)
Scenario (b) Direct SR→HRV effect
  r_raw  = 0.369  (p<0.001, 95% CI 0.31 to 0.42)
  r_part = 0.152  (p<0.001, 95% CI 0.09 to 0.21)
  Δr     = 0.217  (Kp-driven inflation)
Scenario (c) Lagged association  [AR(1), ρ = 0.80]
  r_raw  = 0.148  (p<0.001, 95% CI 0.09 to 0.21)
  r_part = -0.024  (p=0.446, not significant)
  Δr     = 0.124  (Kp-driven inflation)
============================================================
Monte Carlo robustness  (1000 iterations per scenario)
============================================================
Scenario (a) Pure Kp confounder
  r_raw  mean=0.226  SD=0.029
  r_par  mean=0.001  SD=0.031
  Δr     mean=0.201  range=[0.040, 0.282]
Scenario (b) Direct SR→HRV effect
  r_raw  mean=0.392  SD=0.026
  r_par  mean=0.148  SD=0.031
  Δr     mean=0.245  range=[0.177, 0.306]
Scenario (c) Lagged association  [AR(1), ρ = 0.80]
  r_raw  mean=0.158  SD=0.032
  r_par  mean=-0.001  SD=0.032
  Δr     mean=0.133  range=[-0.025, 0.225]
Sensitivity analysis (Kp slope ±50%):
  factor=0.5:  Δr mean=0.049  range=[-0.084, 0.112]
  factor=1.0:  Δr mean=0.201  range=[0.008, 0.284]
  factor=1.5:  Δr mean=0.354  range=[0.189, 0.428]
```

4. Disclaimer

This script uses only synthetic (randomly generated) data. No real participant data are processed. The broadband voltage SD of the SR dataset is NOT the same quantity as calibrated SR modal power; the simulation parameter SR1_INTERCEPT = 7.83 Hz and SR1_KP_SLOPE = −0.05 Hz/Kp represent values from published literature and are used here solely for the purpose of generating a realistic Kp-confounded synthetic dataset.

This code is provided for transparency and reproducibility. The authors take full responsibility for the accuracy of the simulation logic.
